# Lepton flavour violating top decays at the LHC

**DOI:** 10.1140/epjc/s10052-015-3649-5

**Published:** 2015-09-25

**Authors:** Sacha Davidson, Michelangelo L. Mangano, Stéphane Perries, Viola Sordini

**Affiliations:** CERN PH-TH, 1211 Geneva 23, Switzerland; IPNL, CNRS/IN2P3, 4 rue E. Fermi, 69622 Villeurbanne Cedex, France; Université Lyon 1, Villeurbanne, France; Université de Lyon, 69622 Lyon, France

## Abstract

We consider lepton-flavour violating decays of the top quark, mediated by 4-fermion operators. We compile constraints on a complete set of SU(3) $\times$ U(1)-invariant operators, arising from their loop contributions to rare decays and from HERA’s single-top search. The bounds on *e*–$\mu$ flavour change are more restrictive than on $\ell$–$\tau$; nonetheless the top could decay to a jet ${+} e \bar{\mu }$ with a branching ratio of order $10^{-3}$. We estimate that the currently available LHC data (20 fb$^{-1}$ at 8 TeV) could be sensitive to $BR(t \rightarrow e \bar{\mu }$+ jet) ${\sim } 6\times 10^{-5}$, and we extrapolate that 100 fb$^{-1}$ at 13 TeV could reach a sensitivity of ${\sim } 1 \times 10^{-5}$.

## Introduction

Lepton Flavour Violation (LFV) [1–3], meaning local interactions that change the flavour of charged leptons, should occur because neutrinos have mass and mix. This motivates sensitive searches for processes such as $\mu \rightarrow e \gamma$ [4] and $\mu \mathrm{-}e ~\mathrm{conversion}$ [5, 6]. However, the mechanism responsible for neutrino masses is unknown, so it is interesting to parametrise LFV with contact interactions, and to look for it everywhere. In this context, the LHC could have the best sensitivity to LFV processes involving a heavy leg, such as the *Z* [7–31], the Higgs [31–62], or a top [63, 64]. In this paper, we study the LFV top decays $t\rightarrow q e^\pm \mu ^\mp$, where $q \in \{u,c\}$.

We suppose that these decays are mediated by a 4-fermion interaction, and outline in Sect. 2 the current bounds on LFV branching ratios of the top. The bounds arise from rare decays and HERA’s single-top search, and they are discussed in more detail in the appendices. We find that, while these bounds place strong constraints on some specific Lorentz structures for the 4-fermion interactions, they still allow for $t \rightarrow q e^\pm \mu ^\mp$ decays with rates within the LHC reach. In Sect. 3, we estimate the LHC sensitivity to $t\rightarrow q e^\pm \mu ^\mp$, with 20 fb$^{-1}$ of LHC data at 8 TeV. This estimate relies on simulations of the background and signal and is inspired by the CMS search for $t\rightarrow Z q$ [65]. The extrapolation to higher energies and luminosities is discussed in Sect. 4.

*Quark*-flavour-changing top decays, such as $t\rightarrow cZ$ and $t\rightarrow h c$, have been studied in the context of explicit models [62, 66–79] or described by contact interaction parametrisations [80–87], and they have been searched for at the LHC [65, 88–90]. Quark-flavour-changing (but lepton-flavour-conserving) three-body decays of the top, $t \rightarrow c f\bar{f}$, where *f* is a lepton or quark, have also been calculated in explicit models [91–94]. Top interactions that change quark and lepton flavour, and, in addition, baryon and lepton number, have been explored in [95, 96] and searched for by CMS [97]. In models with weak-scale neutrinos *N* [98], there can be lepton number- and flavour-changing *W* decays: $W^- \rightarrow N \ell \rightarrow q \bar{q}' \ell ' \ell$, which could appear in the final state of top decays. In the presence of this decay, $t\bar{t}$ production could give a final state with 3 leptons, missing energy and jets, as in the decay we study (see Fig. 2). However, a different combination of leptons and jets should reconstruct to the top mass. Finally, Fernandez et al. [63] studied almost the same process as us, $t \rightarrow q\tau ^\pm \mu ^\mp$, but mediated by a (pseudo)-scalar boson. They obtained separately the low-energy bounds on the quark- and lepton-flavour-changing couplings of their boson, and they show that LFV top branching ratios can be ${\sim } 10^{-5}$ if the boson mass is ${\lesssim } 2 m_{W}$. Heng et al. [64] calculated LFV top decay rates in R-parity non-conserving supersymmetry, finding rates comparable to our leptoquark estimates.

## Current bounds

We are interested in the decays of a top (or anti-top) to a jet and a pair of oppositely charged leptons of different flavour. In this work, we focus on the processes $t \rightarrow q e^{\pm } \mu ^{\mp }$, where $q\in \{u,c\}$, because the *e* and ${\mu }$ are easy to identify at the LHC, and $e \leftrightarrow \mu$ flavour violation is the most strictly constrained at low energy. We leave the decays to $q e^{\pm } \tau ^{\mp }$ and $q \mu ^{\pm } \tau ^{\mp }$ for a later analysis.

We suppose that these decays are mediated by 4-fermion contact interactions. A complete list of the $SU(3) \times U(1)$ invariant operators that we study is given in Appendix A. We do not impose SU(2) on our operators, because the scale we will probe is not far from the electroweak scale. We refer to these LFV operators as “top operators”. Here, as an example, consider the exchange of a heavy SU(2) singlet leptoquark $S_0$ with couplings $\lambda _{et} S_{0} \overline{e_{R}} t^{c}$ and $\lambda _{\mu {c}} S_{0} \overline{\mu _{R}} c^{c}$, which (after Fierz rearrangement) generates the dimension-six contact interaction

1 $$\begin{aligned}&\frac{\lambda _{et}^*\lambda _{\mu {c}}}{2 m^2_{LQ}} (\overline{\mu }\gamma ^{\alpha } P_{R e})(\overline{c}\gamma ^{\alpha } P_{R t})\nonumber \\&\quad \equiv -\epsilon ^{\mu e ct}_{RR} \frac{4G_F}{\sqrt{2}} (\overline{\mu }\gamma ^\alpha P_{R e})(\overline{c}\gamma ^{\alpha } P_{R t})\nonumber \\&\quad = -\epsilon ^{\mu e ct}_{RR} \frac{4G_{F}}{\sqrt{2}} \mathcal{O}^{RR}_{\mu e ct}. \end{aligned}$$

Alternatively, we could define the operator coefficient as $-1/\Lambda ^2$, in which case $\epsilon \simeq m_t^2/\Lambda ^2$ because $2 \sqrt{2}G_{F} \simeq m_{t}^{-2}$ (we take $m_{t} = 173.3$ GeV). We will quote low-energy bounds on such interactions as limits on the dimensionless $\epsilon$s. In the case of our leptoquark example, $m_{LQ}\gtrsim 1~\hbox {TeV}$ to satisfy current bounds on second generation leptoquarks from the LHC [99–101], and thus, for $\lambda \lesssim 1$, one expects $\epsilon ^{\mu e ct}_{RR} \lesssim 0.02$.

Notice that $t \rightarrow q e^{-} \mu ^{+}$ and $t \rightarrow q e^{+} \mu ^{-}$ are mediated by different operators. Most of the bounds we quote will apply to $|\epsilon ^{\mu e ct}|^{2} +|\epsilon ^{e \mu ct}|^{2}$, and we will study the LHC sensitivity assuming equal rates for $t \rightarrow q e^{-} \mu ^+$ and $t \rightarrow q e^{+} \mu ^{-}$.

### Decay of the top

In the Standard Model, the top decays almost always to $bW^+$, at a tree-level rate given by

2 $$\begin{aligned} \Gamma (t\rightarrow bW)= & {} \frac{g^{2}|V_{tb}|^2m_{t}^{3}}{64\pi m_{W}^{2}} \left( 1-\frac{m_W^2}{m_t^2} \right) ^2 \left( 1+2\frac{m_{W}^{2}}{m_{t}^{2}}\right) \nonumber \\\simeq & {} 1.3~\mathrm{GeV}. \end{aligned}$$

In the presence of the operator of Eq. (1), the three-body decay rate is

3 $$\begin{aligned} \Gamma (t\rightarrow e^{+} \mu ^{-} + c) = |\epsilon ^{\mu e ct}_{RR}|^{2} \frac{G_{F}^{2} m_{t}^{5}}{192\pi ^{3}}, \end{aligned}$$

so the branching ratio, allowing for all the operators listed in Appendix A, and neglecting fermion masses other than the top (to remove interferences), is

4 $$\begin{aligned}&BR (t \rightarrow \ell _{i}^{+} \ell _{j}^{-} + q)\nonumber \\&\quad \simeq \frac{1.3}{48\pi ^2}{\Big (}|\epsilon ^{ijqt}_{LL}|^2 + |\epsilon ^{ijqt}_{LR}|^2 + |\epsilon ^{ijqt}_{RL}|^2 + |\epsilon ^{ijqt}_{RR}|^2\nonumber \\&\qquad + \frac{1}{4} {\Big [}|\epsilon ^{ijqt}_{S+P,R}|^2 + |\epsilon ^{ijqt}_{LQ,R}|^2 -2 \mathrm{Re}\{\epsilon ^{ijqt}_{S+P,R} \epsilon ^{ijqt *}_{LQ,R}\} {\Big ]}\nonumber \\&\qquad + \frac{1}{4} {\Big [}|\epsilon ^{ijqt}_{S+P,L}|^2 + |\epsilon ^{ijqt}_{LQ,L}|^2 - 2\mathrm{Re}\{ \epsilon ^{ijqt}_{S+P,L} \epsilon ^{ijqt *}_{LQ,L}\} {\Big ]}\nonumber \\&\qquad + \frac{1}{4} {\Big [}|\epsilon ^{ijqt}_{S-P,L}|^2x + |\epsilon ^{ijqt}_{S-P,R}|^2 {\Big ]} {\Big )}. \end{aligned}$$

Here $q \in \{ u,c\}$, and we approximated $y_t |V_{tb}|\simeq 1$. This is small ($\frac{1.3}{48\pi ^2 } \simeq 2.8 \times 10^{-3}$), due to the three-body phase space. For the leptoquark example discussed above, $BR(t \rightarrow c e^+ \mu ^-) \simeq 10^{-6}$ for $\epsilon ^{\mu e ct}_{RR} = 0.02$.

The phase space distribution of the $\ell _i^+ \ell _j^-$ and *q* depends on the Lorentz and spinor structure of the contact interaction and could affect the efficiency of experimental searches for this decay. The squared matrix elements for the individual contact interactions have the form $|\mathcal{M}|^2 \propto x(1-x)$, where $x = m_{ab}^2/m_t^2$ and $m_{ab}^2$ is the invariant mass-squared of a pair of final state fermions *a* and *b*. Our study will not take this into account, since we found, in some explicit examples, only a small relative effect on the selection efficiency (of the order of 5 %).

### Bounds from flavour physics and HERA

Low-energy constraints on 4-fermion operators involving 2 leptons and 2 quarks have been estimated and compiled for many operators taken one at a time [102–104], and carefully studied for selected flavour combinations (see e.g. [105], or global fits to $b\overline{s} \mu \overline{\mu }$ operators [106–109]). However, even in the more recent compilations [103, 104], bounds on LFV operators involving a single top are not quoted. In Appendices B and C, we estimate bounds on such operators from their possible contributions, inside a loop, to rare $\mu$, *B* and *K* decays. In Appendix D, we estimate bounds from single-top searches at HERA. Here, we summarise the resulting bounds, and we list in Tables 1 and 2 the best limits on the coefficients of the various operators. We will find that only the coefficients of some operators are stringently constrained, while others could mediate LFV top decays within the sensitivity of the LHC.

The current upper limit $BR(\mu \rightarrow e \gamma ) < 5.7\times 10^{-13}$ [4] severely restricts $e\leftrightarrow \mu$ flavour change. For our top operators to contribute, the quark lines must be closed, which requires at least two loops and a CKM factor, see the second diagram of Fig. 5. Nonetheless, in the case of scalar or tensor operators involving $t_R$ this diagram can overcontribute to $\mu \rightarrow e \gamma$ by several orders of magnitude, because the lepton chirality flip is provided by the operator (rather than $m_\mu$), so the diagram is enhanced by a factor $m_t/m_\mu$. We make order-of-magnitude estimates in Appendix B, and we quote the resulting bounds in Table 2.

**Table 1:** Constraints on the dimensionless coefficient $\epsilon _{XY}^{ijqt}$ of the 4-fermion operator $\mathcal{O}^{XY}_{ijqt}=2 \sqrt{2} G_F (\bar{e}_{i} \gamma ^{\mu } P_{X} e_{j})(\bar{u}_{q}\gamma _{\mu } P_{Y} t)$ (see the operator list in Appendix A), where *XY* are given in the top row, and generation indices *ijqt* are given in the first column. The bounds are on the first line of each box, their origin on the second. They can arise from the HERA single-top search, or from the loop contribution to the operator involving lighter fermions whose coefficient is given in parentheses below the bound (see Appendix C.1 for current experimental bounds on the lighter-fermion operator coefficients). The $\sim$ bounds are discussed in Appendix D.4. We expect that $\epsilon < 1$, so in boldface are the bounds that impose an upper limit smaller than 1

*ijqt*	*LL*	*RL*	*LR*	*RR*
$e\mu ut$	$\mathbf{0.0037}$	$\mathbf{0.0037}$	$\mathbf{0.33}$	$\mathbf{0.22}$
	$(\epsilon ^{e\mu ds}_{LL})$	$(\epsilon ^{e\mu ds}_{LL})$	(HERA)	(HERA)
$e\mu ct$	$\mathbf{0.015}$	$\mathbf{0.015}$	1	1
	$(\epsilon ^{e\mu ds}_{LL})$	$(\epsilon ^{e\mu ds}_{LL})$	$(\epsilon ^{e\mu ds}_{LL})$	$(\epsilon ^{e\mu ds}_{LL})$
$e\tau ut$	1.2	1.2	${\sim } 1.3$	${\sim } 0.85$
	$(\epsilon ^{e\tau db}_{LL})$	$(\epsilon ^{e\tau db}_{LL})$	(HERA)	(HERA)
$e\tau ct$	1	1	60	60
	$(\epsilon ^{ e\tau sb}_{LL})$	$(\epsilon ^{e\tau sb}_{LL})$	$(\epsilon ^{e\tau sb}_{LL})$	$(\epsilon ^{e\tau sb}_{LL})$
$\mu \tau ut$	0.8	0.8	$-$	$-$
	$(\epsilon ^{ \mu \tau db}_{LL})$	$(\epsilon ^{\mu \tau db}_{LL})$		
$\mu \tau ct$	1.5	1.5	100	100
	$(\epsilon ^{ \mu \tau sb}_{LL})$	$(\epsilon ^{\mu \tau sb}_{LL})$	$(\epsilon ^{ \mu \tau sb}_{LL})$	$(\epsilon ^{\mu \tau sb}_{LL})$

**Table 2:** Constraints on the dimensionless coefficient $\epsilon ^{ijqt}$, of the scalar and tensor 4-fermion interactions. See Appendix A for operator definitions corresponding to the subscript of $\epsilon$. The generation indices *ijqt* are given in the first column. Beneath each bound is given its origin in parentheses; $\epsilon ^{ij \alpha \beta }_{S \pm P,X}$ are the limits of Table 7, and $\epsilon ^{i \nu q \beta }_{CC}$ are from Table 8. See the caption of Table 1 for additional details

*ijqt*	$\epsilon _{S - P,R}$	$\epsilon _{S + P,R}$	$\epsilon _{T,R}$	$\epsilon _{S-P,L}$	$\epsilon _{S+P,L}$	$\epsilon _{T,L}$
$e\mu ut$	$\mathbf {\mathcal{{O} }( 10^{-2})}$	$\mathbf {\mathcal{{O}} ( 10^{-2})}$	$\mathbf {\mathcal{{O}} ( 10^{-2})}$	$\mathbf {0.66}$	$\mathbf { 0.03}$	
	$(\mu \rightarrow e \gamma )$	$(\mu \rightarrow e \gamma )$	$(\mu \rightarrow e \gamma )$	HERA	$(\epsilon ^{e\nu u b}_{CC})$	
$e\mu ct$	$\mathbf {\mathcal{{O}} ( 10^{-3})}$	$\mathbf { \mathcal{{O}} (10^{-3})}$	$\mathbf {\mathcal{{O}} ( 10^{-3})}$		22	
	$(\mu \rightarrow e \gamma )$	$(\mu \rightarrow e \gamma )$	$(\mu \rightarrow e \gamma )$		$(\epsilon ^{e\nu cs}_{CC})$	
$e\tau ut$	23	23			$\mathbf { 0.03}$	
	$(\epsilon ^{e\tau db}_{S \pm P,X})$	$(\epsilon ^{e\tau db}_{S \pm P,X})$			$(\epsilon ^{e\nu ub}_{CC})$	
$e\tau ct$	100	100			22	
	$(\epsilon ^{e\tau db}_{S \pm P,X})$	$(\epsilon ^{e\tau db}_{S \pm P,X})$			$(\epsilon ^{e\nu cs}_{CC})$	
$\mu \tau ut$	21	21			$\mathbf { 0.03}$	
	$(\epsilon ^{\mu \tau db}_{S \pm P,X})$	$(\epsilon ^{\mu \tau db}_{S \pm P,X})$			$(\epsilon ^{\mu \nu ub}_{CC})$	
$\mu \tau ct$	100	100			100	
	$(\epsilon ^{\mu \tau db}_{S \pm P,X})$	$(\epsilon ^{\mu \tau db}_{S \pm P,X})$			$(\epsilon ^{\mu \nu cs}_{CC})$	

**Fig. 1:**
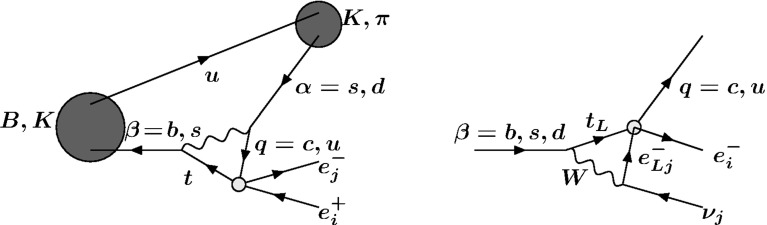
Diagrams for generating an LFV operator with light external quark legs, by dressing a top operator with a *W* loop. To reduce index confusion, down-type quarks have Greek indices

Exchanging a *W* between the *t* and *q* quark legs of the top operator will generate an operator with down-type external quark legs, see the left diagram of Fig. 1. The coefficient of this light quark operator will be suppressed by a loop, CKM factors, and various masses. Numerical values for these suppression factors are given in Table 9 of the appendix; however, their approximate magnitude is simple to estimate. If the top is singlet ($t_R$), then the loop is finite; in the case of $V \pm A$ interactions, this is because mass insertions are required on both internal quark lines to flip chirality. In the case of scalar operators, one internal quark mass for chirality flip is still required; then terms linear in the loop momentum vanish, so the diagram is also proportional to an external quark mass. For scalar operators involving $t_L$ (which require an $m_q$ insertion to connect the *W* to the *q* line), the best limit can arise from exchanging a *W* between the *t* and a charged lepton leg, which generates a charged-current operator as represented in the second diagram of Fig. 1. In the appendix are also given current bounds on the coefficients of the various light quark operators that the top operators can induce. Comparing these bounds to the induced coefficients, gives the limits of Tables 1 and 2 that are labelled with ($\epsilon$)s.

HERA collided protons with positrons (or electrons) at a centre-of-mass energy of 319 GeV, and searched for single tops in the final state. The H1 collaboration had a few events with energetic isolated leptons and missing energy, consistent with $e^\pm p\rightarrow te^\pm + X$ followed by leptonic decay of the top [110, 111]. However, this signal was not confirmed by the ZEUS experiment [112], and neither collaboration had a signal consistent with hadronic top decays. Both collaborations set bounds on $\sigma (e^\pm p\rightarrow e^\pm tX)$; we follow H1, since they had some events and a weaker bound:

5 $$\begin{aligned} \sigma (e^\pm p\rightarrow e^\pm t +X) \le 0.30~\hbox {pb}= & {} \frac{2.3\times 10^{-5}}{m_t^2} \quad \hbox {at }95~\% \hbox { CL}.\nonumber \\ \end{aligned}$$

Contact interactions of the form $(\overline{e} \Gamma \mu ) (\overline{u} \Gamma t )$ and $(\overline{\mu } \Gamma e ) (\overline{u} \Gamma t )$ could, respectively, mediate $e^-p\rightarrow \mu ^- t X$ and $e^+p\rightarrow \mu ^+ t X$. As discussed in Appendix D, the limit of Eq. (5) translates to $\epsilon \lesssim 0.3 \rightarrow 1$ for the various operators, as given in Tables 1 and 2.

It can be seen from Tables 1 and 2, that rare decays give very weak bounds on some contact interactions of the form $(\overline{e} \Gamma \tau ) (\overline{u} \Gamma t )$ and $(\overline{\tau } \Gamma e ) (\overline{u} \Gamma t )$. Such interactions might have contributed a signal at HERA via leptonic $\tau$ decays, so we make some approximate estimates in Appendix D.4, and we include the bounds in the tables with a $\sim$.

### Implications

The current bounds on LFV branching ratios of the top can be obtained from Tables 1 and 2, and from and Eq. (4). In these tables, the bound on $\epsilon ^{ijqt}_{XYZ}$ appears on the first row of each box, where the flavour indices *ijqt* are given in the left column, and the operator label *XYZ* is given in the first row (see Appendix A for the operator definitions). In parentheses below the bound is a clue to where it comes from: HERA means the single-top searches at HERA that are discussed in Appendix D, and the ($\epsilon$)s mean the bound comes from dressing the top operator with a *W* loop. For instance, the bound 0.0037 in the second row and third column of Table 1 arises by exchanging a *W* between the *t* and *u* legs of the top operator, which gives the operator $(\overline{e} \gamma ^\rho P_L \mu ) (\overline{d} \gamma _\rho P_Ls )$. This would mediate the unobserved decay $K^0 \rightarrow e\mu$, so its coefficient is bounded above, as indicated in Table 6. (The bounds on lighter-quark operators relevant to constraining top operators are given in Tables 6, 7 and 8, and Table 9 gives the loop suppression factors with which the top operators generate the lighter-quark operators.) Translated back to the top operator, the upper limit on $BR(K^0 \rightarrow e\mu )$ gives the quoted limit on the top-operator coefficient.

In this paper, we are interested in top decays to $e^\pm \mu ^\mp$, the bounds on which are given in the second and third rows of the tables. For many^1^ of the operators involving the doublet component of the top ($t_L$; recall that the last index in the operator label is the top chirality), the rare decay bounds are restrictive, implying that these operators could only induce $BR(t\rightarrow q \mu ^\pm e^\mp ) \le 10^{-6}$. Scalar and tensor operators involving *e*, $\mu$ and $t_R$ would overcontribute to $\mu \rightarrow e \gamma$. However, there remain operators which are weakly or not constrained, allowing for a branching ratio ${\lesssim } 10^{-3}$. It is therefore interesting to explore the sensitivity of the LHC to $t\rightarrow e^\pm \mu ^\mp q$ decays.

Finally, it is interesting to consider how large the $$\epsilon $$ coefficient of the top operators can be. Some of the upper bounds quoted in Tables 1 and 2 are $$\gg $$1, and should not be interpreted as relevant constraints.^2^ Indeed, the width of the top is given by D0 [113] as $$2.0 \pm 0.5$$ GeV (the theoretical decay rate to *bW* is 1.3 GeV), which constrains $$\epsilon ^{ijqt}_{XYZ} < 10{-}20$$. Furthermore, phenomenological prejudice and the leptoquark example of Eq. (1), suggest that $$\epsilon <1$$, because the three-body decay should be mediated by sufficiently heavy ($$m>m_t)$$ particles, with sufficiently small couplings to have not yet been detected. We therefore quote in boldface the “relevant” bounds that impose $$\epsilon < 1$$.

## ${t\rightarrow e^\pm \mu ^\mp q}$ at the 8 TeV LHC

**Fig. 2:**
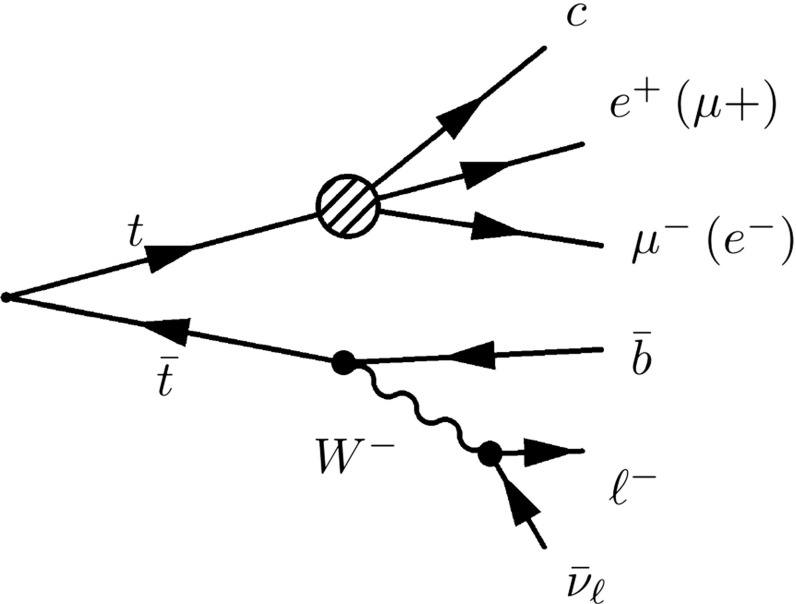
Feynman diagram for the considered signal where $\ell =e$ or $\mu$ (the conjugate diagram is also considered)

In this section, we estimate the sensitivity of current LHC data to the LFV top decays $t \rightarrow q e^{\pm }\mu ^{\mp }$, where $q=u, c$. We consider strong production of a $t\bar{t}$ pair, because this is the most abundant source of tops at the LHC, followed by the leptonic decay on one top, and the LFV decay of the other. This is illustrated in Fig. 2 and gives a final state containing 3 isolated muons or electrons,^3^ which has small Standard Model backgrounds.

### Simulation setup

This study is performed for proton–proton collisions at the LHC, with a centre-of-mass energy of 8 TeV and an integrated luminosity of 20 $\mathrm {fb}^{-1}$, corresponding to the LHC Run1. The details of the signal and background generation are given in Sect. 3.2. The detector simulation is carried out by Delphes [114] using a CMS setup parametrisation.

Delphes uses a particle-flow-like reconstruction. The relative isolation of leptons is calculated from the total $p_T$ of the particles inside a cone of $\Delta R$ around the lepton direction ($\Delta R=0.3$ for electrons and 0.4 for muons), divided by the $p_T$ of the lepton. Jets are clustered using the fastjet package [115] with the Anti-kt [116] algorithm with distance parameter $R=0.5$. The b-tagging performances are tuned on the typical efficiency and fake rate obtained in CMS.

For this study, no additional interactions in the same or neighbouring bunch crossing (pileup) are simulated.

### Signal and SM backgrounds generation

The signal is generated with PYTHIA 8.205 [117] using tune 4C. Top quarks are pair produced, then one top is forced to decay to charm, $\mu ^\pm$, and $e^\mp$, with equal probability between $\mu ^+ \, e^-$ and $\mu ^- \, e^+$. The decay products are distributed according to the available phase space. 100k events have been generated both for LFV top and anti-top decays.

The backgrounds for this search, listed in Table 3, are processes that can give rise to 3 isolated leptons and at least 2 jets in the final state. Most of them are related to the production of real isolated leptons, e.g. from a top pair or vector bosons in the final state. In the table, are also shown the details as regards the number of generated events and production cross section for 8 and 13 TeV proton–proton collisions. The number of generated events refers to the generation at 8 TeV. The $t\bar{t}$ cross section is calculated with the Top++2.0 program to next-to-next-to-leading order in perturbative QCD, including soft-gluon resummation to next-to-next-to-leading-log order (see [118] and references therein), and assuming a top-quark mass of $m_{t} = 173.3$ GeV. When an explicit calculation was not available, the cross sections have been calculated with the MCFM package [119], version 7.0. The kinematic cuts used for the calculation are also shown in the table.

**Table 3:** Number of events generated, and cross sections at NLO (except for $t\bar{t}{+}$jets), for each background category. Here $l=e, \mu , \tau$

Process	Events	$\sigma$ [pb] (8 TeV)	$\sigma$ [pb] (13 TeV)	Source
$t\bar{t}$ (2l2$\nu$2b)	12M	26.19	86.26	Top++2.0 (NNLO) [118]
WW+jets (2l2$\nu$, $m_{ll}>10~\hbox {GeV}/\hbox {c}^2$)	1M	5.84	11.57	MCFM [120, 121]
ZZ+jets (2l2q, $m_{ll}>10~\hbox {GeV}/\hbox {c}^2$)	2.5M	2.71	5.35	MCFM [120, 121]
ZZ+jets (2l2$\nu$, $m_{ll}>10~\hbox {GeV}/\hbox {c}^2$)	1M	0.774	1.53	MCFM [120, 121]
ZZ+jets (4l, $m_{ll}>10~\hbox {GeV}/\hbox {c}^2$)	2.5M	0.390	0.738	MCFM [120, 121]
WZ+jets (2l2q, $m_{ll}>10~\hbox {GeV}/\hbox {c}^2$)	1M	2.37	4.60	MCFM [120, 121]
WZ+jets (3l$\nu$, $m_{ll}>10~\hbox {GeV}/\hbox {c}^2$)	1M	1.15	2.23	MCFM [120, 121]
$t\bar{t}$W+jets	1M	0.212	0.612	MCFM [120, 121]
$t\bar{t}$Z+jets	1M	0.192	0.798	aMC@NLO [122]
tbZ+jets	2M	0.014	0.047	aMC@NLO [122]

The leading order (LO) matrix element generator, MADGRAPH 5 [123], with CTEQ6 parton distribution functions, is used to generate top pair production, and associated production of a top pair and a vector boson (*ttW*, *ttZ*). MADGRAPH, interfaced with tauola for $\tau$ decays, is used to generate vector–vector production (*WW*, *WZ* and *ZZ*) and the contribution of weak processes giving rise to final states with one top quark, one *b* quark and a *Z* boson (decaying to leptons). For the vector–vector production, we only consider final states with at least 2 real charged leptons. This means that for the *WW* system, the considered final states are 2 charged leptons and 2 neutrinos; for *WZ*, they are 3 charged leptons and one neutrino or 2 charged leptons and 2 quarks; and for *ZZ*, they are 2 charged leptons and 2 neutrinos, 2 charged leptons and 2 quarks, or 4 charged leptons. In all cases, MADGRAPH accounts for the presence of up to 2 additional jets at matrix-element level, and the hadronisation is carried out by PYTHIA 8.205. The details of the SM background simulation and cross sections are shown in Table 3.

### Event selection

The signal for this search is $t\bar{t}$ production, followed by the lepton-flavour violating decay of one top (which will be denoted as LFV top in the following), and the leptonic decay of the other (standard top, in the following). The number of expected signal events is given by

6 $$\begin{aligned} N_{\mathrm{SIG}}={{\mathcal {L}}}\cdot \epsilon _{\mathrm{SIG}}\cdot 2\cdot \sigma _{t\bar{t}}\cdot BR(W\rightarrow l\nu ) \cdot BR(t\rightarrow q \mu ^\pm e^\mp ),\nonumber \\ \end{aligned}$$

where $l \in \{e,\mu ,\tau \}$, ${\mathcal {L}}$ the integrated luminosity, $\epsilon _{\mathrm{SIG}}$ the selection efficiency on the signal, and $\sigma _{t\bar{t}}=246.7$ pb (see [118] and references therein).

The considered signature is 3 isolated leptons (with one pair of opposite sign and opposite flavour from the LFV decay), 2 jets (one of which is a *b*-jet), and missing transverse energy. For the event selection, we consider only muons of $p_T>20$ GeV and $|\eta |<2.4$, electrons of $p_T>20$ GeV and $|\eta |<2.5$, and jets of $p_T>30$ GeV and $|\eta |<2.4$. These criteria are comparable to those used in real analyses by the CMS or ATLAS collaborations. A muon is considered isolated if its relative isolation value is less that 0.12, and an electron is considered isolated if its relative isolation value is less that 0.1. We select events containing:

1. exactly 3 isolated charged leptons (electrons or muons), 2 of which must be of opposite sign and opposite flavour.
2. Events are requested to contain at least 2 jets, and
3. exactly one b-tagged jet, and
4. the missing transverse energy has to be higher than 20 GeV.
5. In order to exclude events where 2 of the isolated leptons come from a real Z boson, we reject events containing any pair of opposite sign isolated muons or electrons with invariant mass between 78 and 102 GeV/c$^2$. This cut is particularly helpful in rejecting background arising from $t\bar{t}$ associated production with a Z.
6. The charged lepton that does not belong to the pair of opposite sign and opposite flavour leptons is assigned to the standard top in the event, and assumed to come from the W decay (bachelor lepton). Following a common procedure in reconstruction of $t\bar{t}$ semi-leptonic events, the *x* and *y* components of the missing transverse energy are taken as a measurement of the neutrino $p_{x}$ and $p_{y}$, and the longitudinal component of the neutrino momentum is calculated imposing the requirement that the invariant mass of the system composed of the bachelor lepton and the neutrino must equal the mass of the W boson. The bachelor lepton and the neutrino 4-momenta are then combined with that of the b-tagged jet, to build a candidate standard top. When more choices of the bachelor lepton are possible (there can be up to two possible pairs of opposite sign opposite flavour charged leptons in one event), all are considered and the one giving the best standard top mass is chosen. We reject events in which the invariant mass of the standard top candidate is more than 45 GeV away from the nominal top mass. After the choice of the bachelor lepton, there is only one possible pair of opposite sign and opposite flavour leptons in each event. This is combined with all good (non b-tagged) jets present in the event to build a list of candidates for the LFV top. Events are required to have at least one combination giving a LFV top mass within 25 GeV of the nominal value.

The efficiency of the final selection on signal events is

7 $$\begin{aligned} \epsilon _{\mathrm{SIG}}= (1.85\pm 0.03)~\%, \end{aligned}$$

where the uncertainty is statistical only. The signal efficiency is calculated on $t\bar{t}$ events where one top decays through $t\rightarrow q \mu ^\pm e^\mp$, and the other one decays to a *b* quark and a *W*, which subsequently decays to a charged lepton (e, $\mu$ or $\tau$) and a neutrino, and is defined as the fraction of such events passing the selection criteria.

The number of expected events, for the signal and for each background category, on 20 fb$^{-1}$ of proton–proton data at 8 TeV is shown in Table 4, for different subsequent selection requirements.

**Table 4:** Number of expected events for a luminosity of 20 fb$^{-1}$, at various steps of the selection, for the signal process normalised to a branching ratio $BR(t\rightarrow qe\mu )=6.3 \times 10^{-5}$, and the various backgrounds considered in this study normalised to their NLO cross sections. All uncertainties are statistical only. The considered backgrounds are the same as in Table 3, grouped in wider categories. In particular, the numbers in the “no selection” column are relative only to the final states detailed in Table 3. The steps in the selection are as follows; step 1: 3 leptons with 2 opposite sign opposite flavour, step 2: at least 2 jets, step 3: exactly one b-tag, step 4: missing $E_T$ greater than 20 GeV, step 5: Z boson veto, step 6: invariant masses cuts (see Sect. 3.3 and Fig. 3). Uncertainties are statistical only

Process	No selection	Step 1	Step 2	Step 3	Step 4	Step 5	Step 6
Signal	202.57	$32.98 \pm 0.17$	$22.66 \pm 0.14$	$9.12 \pm 0.09$	$8.20 \pm 0.09$	$7.50 \pm 0.09$	$3.75 \pm 0.06$
$t\bar{t}$	542806	$14.78 \pm 0.81$	$10.51 \pm 0.69$	$4.36 \pm 0.44$	$4.36 \pm 0.44$	$3.55 \pm 0.40$	$0.63 \pm 0.17$
WW+jets	116760	$0.93 \pm 0.33$	$0.35 \pm 0.20$	${<} 0.35$	${<} 0.35$	${<} 0.35$	${<} 0.35$
ZZ+jets	72900	$353.74 \pm 0.95$	$82.50 \pm 0.47$	$3.74 \pm 0.10$	$1.60 \pm 0.07$	$0.25 \pm 0.03$	$0.03 \pm 0.01$
WZ+jets	63360	$852.21 \pm 4.04$	$182.96 \pm 1.90$	$8.70 \pm 0.42$	$7.62 \pm 0.39$	$0.74 \pm 0.12$	$0.04 \pm 0.03$
$t\bar{t}$W	4240	$9.36 \pm 0.24$	$7.67 \pm 0.22$	$3.59 \pm 0.15$	$3.45 \pm 0.14$	$3.10 \pm 0.14$	$0.27 \pm 0.04$
$t\bar{t}$Z	3840	$17.25 \pm 0.33$	$16.44 \pm 0.32$	$7.72 \pm 0.22$	$7.16 \pm 0.21$	$1.85 \pm 0.11$	$0.22 \pm 0.04$
tbZ	282	$5.75 \pm 0.03$	$3.59 \pm 0.02$	$1.51 \pm 0.01$	$1.37 \pm 0.01$	$0.13 \pm 0.01$	$0.01 \pm 0.01$

### Results and expected limits on the branching ratio

The selection and its efficiency, on signal and background, are discussed in Sect. 3.3, and summarised in Table 4. Assuming a branching ratio of $BR(t\rightarrow q \mu ^\pm e^\mp )=6.3 \times 10^{-5}$ for the signal, an uncertainty of 2.5 % on the luminosity, and 20 fb$^{-1}$ of data, we would expect $N_{\mathrm{SIG}}=3.75\pm 0.06$ signal events, to compare to the $N_{\mathrm{BKG}}=1.20\pm 0.18$ expected events from known backgrounds.

**Fig. 3:**
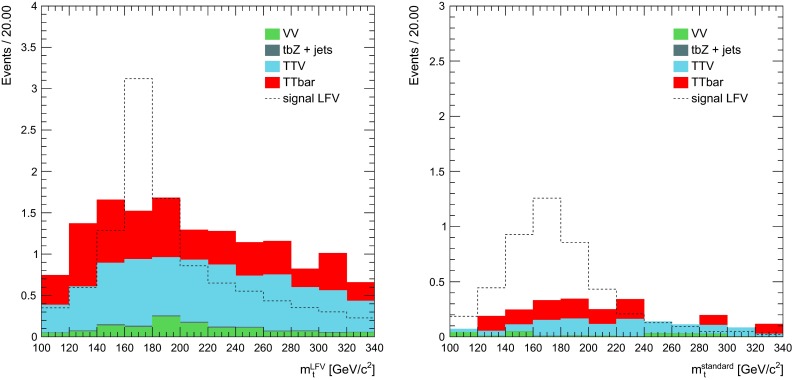
Invariant mass of the LFV top candidates (*left*) and standard top candidate (*right*) in events passing all the selection, apart from the cut on the masses themselves. The different background contributions are shown in filled histograms and stacked, normalised to the number of events expected in 20 fb$^{-1}$. For comparison, the distribution for signal events is also shown as a *dashed line*, normalised to the number of events expected in 20 fb$^{-1}$, for an example signal branching ratio $BR(t\rightarrow q \mu ^\pm e^\mp ) = 6.3 \times 10^{-5}$, equal to the expected limit extracted in Sect. 3.4

In Fig. 3, we show the invariant mass of the LFV top candidate (left) and the standard top candidate (right), in events passing all the cuts except those on the masses themselves.

In order to evaluate the sensitivity of this search for $t\rightarrow q \mu ^\pm e^\mp$, we calculate the expected upper limit that could be set, in the case of absence of the signal. The calculation is based on the number of expected background events surviving the final selection, in 20 fb$^{-1}$ of 8 TeV LHC data, so the result can be interpreted as the possible upper limit the CMS or ATLAS collaborations (alone) could expect to set with Run1 data, if the signal were not there.

A 95 $\%$ confidence level (CL) upper limit on the branching fraction of $t\rightarrow q \mu ^\pm e^\mp$ is calculated using the modified frequentist approach (CL$_{s}$ method [124, 125]), as it is implemented in the RooStats framework [126]. Based on the number of expected background events, summarised in Table 4, and on Eq. (6), the obtained limit is

$$\begin{aligned} BR(t\rightarrow q \mu ^\pm e^\mp ) < 6.3 \times 10^{-5}\quad \hbox {at }95~\%\hbox { CL}. \end{aligned}$$

Alternative techniques for limit calculations, as implemented in RooStats, have been tried, leading to compatible results. An eventual variation of 100 $\%$ in the number of expected background events would lead, in the worst case, to an expected limit of $BR(t\rightarrow q \mu ^\pm e^\mp ) < 7.4 \times 10^{-5}$ at 95 % CL.

**Table 5:** Expected upper limits on $BR(t\rightarrow q \mu ^\pm e^\mp )$, under the hypothesis of the absence of signal, for 8, 13 TeV (in two scenarios: the case of 20 and 100 fb$^{-1}$ collected luminosity) and 14 TeV for 3000 fb$^{-1}$ collected luminosity

	8 TeV (20 fb$^{-1}$)	13 TeV (20 fb$^{-1}$)	13 TeV (100 fb$^{-1}$)	14 TeV (3000 fb$^{-1}$)
$BR(t\rightarrow q \mu ^\pm e^\mp )$	${<} 6.3\times 10^{-5}$	${<} 2.9\times 10^{-5}$	${<} 1.2\times 10^{-5}$	${\lesssim } 2 \times 10^{-6}$

As explained in Appendix E, we emulate in our framework the published CMS search for $BR(t\rightarrow Z q)$ [65]. The reason for this exercise is twofold: on one hand it allows one to validate our procedure on simulated samples, by comparing with the CMS background expectations. On the other hand, it provides an estimate of the constraint set on $BR(t\rightarrow q \mu ^\pm e^\mp )$ from this previous analysis, which is found to be $BR(t\rightarrow q \mu ^\pm e^\mp )< 3.7\times 10^{-3}$, on the verge of probing LFV top decays mediated by a 4-fermion operator. As proven in the present study, a dedicated analysis would set a limit 50 times stronger (of the order of $BR(t\rightarrow q \mu ^\pm e^\mp ) < 6.3 \times 10^{-5}$), showing that the existing LHC data from Run1 can still be used to obtain interesting constraints on lepton-flavour violation.

## Discussion

### Perspectives at 13 and 14 TeV

To estimate the reach of the described search at a centre-of-mass energy of 13 TeV, we extrapolate the 8 TeV results, rather than performing a full simulation of signal and background processes at 13 TeV. The increase of the production cross sections for SM processes, from 8 to 13 TeV (see Table 3), is taken into account. The selection requirements and efficiencies are kept the same as for the 8 TeV analysis. For the signal, we have checked on simulated events that the efficiencies at 8 and 13 TeV are consistent within 5 %.

The sensitivity is estimated by calculating the expected upper limits on $BR(t\rightarrow q \mu ^\pm e^\mp )$, in the absence of signal, for two scenarios: the case of 20 and 100 fb$^{-1}$ proton–proton data collected by the LHC at 13 TeV centre-of-mass energy. We also extrapolate the sensitivity to the case of 3000 fb$^{-1}$ of integrated luminosity at 14 TeV. In this last case, we simply rescale signal and background rates from 13 to 14 TeV, and use the square root of the number of expected background events as an estimate of their uncertainty. The obtained values are summarised in Table 5. The upper limits presented here are derived using statistical uncertainties only, so do not take into account the possibility for such analyses to become systematically dominated in the future. In order to have an accurate evaluation of the systematics evolution, a deeper study from the LHC experiments would be needed. On the other hand, for an analysis on 13 or 14 TeV data, the selection would have to be re-optimised, possibly leading to an increase in sensitivity.

### Single top

**Fig. 4:**
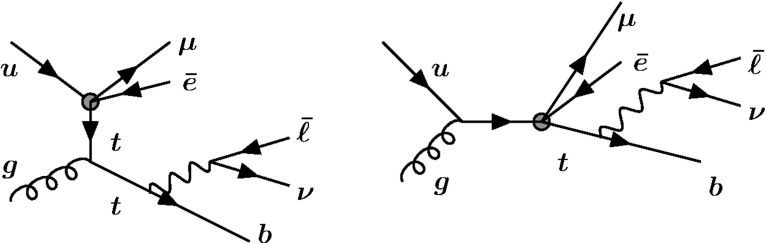
Single-top production via the LFV contact interaction, which produces a final state similar to the considered signal

**Fig. 5:**
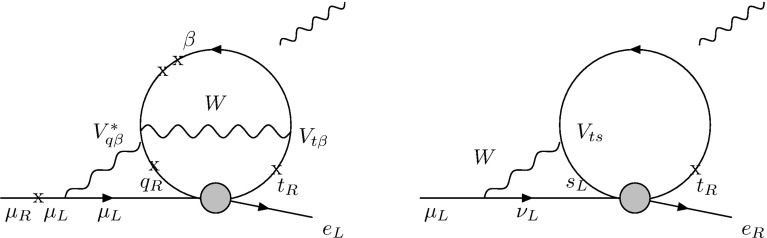
*Left* Example three-loop diagram by which a the neutral current vector operator of Eq. (24) could induce $\mu \rightarrow e \gamma$. “x” is a mass insertion, the *grey blob* is the operator, and the photon could be attached to any quark line. *On the right* is an example two-loop diagram by which a tensor/scalar operator could induce $\mu \rightarrow e \gamma$ without GIM suppression and with $m_t/m_\mu$ enhancement

In addition to mediating LFV top decays, the top operators listed in Appendix A could lead to single-top production with an $e^\pm \mu ^\mp$ pair, as illustrated in Fig. 4. The objects in the final state would be the same as for the $t\bar{t}$ process we studied: 3 leptons, missing energy, and ${\ge } 2$ jets, of which one is a *b*. We estimate that at 8 TeV, the cross section for $pp \rightarrow e^\pm \mu ^\mp t\rightarrow e^\pm \mu ^\mp , \overline{\ell } \nu b$ is of similar order to the cross section for $pp \rightarrow \bar{t} t \rightarrow e^\pm \mu ^\mp \bar{q} , \overline{\ell } \nu b$, for operators involving a *u* quark and slightly less for a *c* quark.

We neglect this process for two reasons. First, the contact interaction approximation (the |4-momentum$|^2$ in the process ${\ll }\Lambda ^2$) is more difficult to justify than in top decay, because the energy scale in the process can be ${\gg }m_t$. Second, finding such events in the backgrounds could be more challenging because the $e^\pm \mu ^\mp$ cannot be required to participate in the reconstruction of a top (step 6 of Sect. 3.3).

We envisage that it makes sense to neglect the LFV single top process in a first search for LFV top decays. This is conservative, because LFV single-top production could contribute events that pass our selection. In the absence of a signal, such a search could sufficiently constrain the contact interaction scale $\Lambda$, to justify including the single top process in subsequent analyses.

## Summary

The aim of this paper was to explore the LHC sensitivity to the decay $t\rightarrow q \mu ^\pm e^\mp$, which is lepton and quark flavour-changing, but baryon and lepton number conserving. We parametrise this decay as occurring via a contact interaction, and list a complete set of $SU(3)\times U(1)$ invariant dimension-six operators in Appendix A. We parametrise the coefficient of these interactions, which we refer to as “top operators”, as $\epsilon 2\sqrt{2} G_F$ or equivalently $1/\Lambda ^2$, with

$$\begin{aligned} \epsilon \simeq \frac{m_t^2}{\Lambda ^2}. \end{aligned}$$

Model-building prejudice (see Sect. 2.3) suggests that $\epsilon \lesssim 1$. The top branching ratio is then

$$\begin{aligned} BR(t\rightarrow q \ell _i^+ \ell _j^-) \simeq 1.3 \frac{|\epsilon |^2}{48\pi ^2} \times \left\{ \begin{array}{cc} 1 &{} V\pm A\\ \frac{1}{4} &{} S\pm P. \end{array} \right. \end{aligned}$$

These contact interactions are currently constrained from their contribution in loops to rare decays, and from single top searches at HERA. These bounds are discussed in the appendices, and summarised in Sect. 2. For interactions involving *e* and $\mu$, rare decay bounds impose $\epsilon \lesssim 0.01$ for some operators, but others can have $\epsilon \sim 1$.

In Sect. 3, we evaluate the sensitivity reach of a dedicated search for lepton-flavour violation in top decays, at the 8 TeV LHC. The search targets $t\bar{t}$ events, where one top decays to an up-type quark (*u* or *c*) and a pair of leptons of opposite sign and opposite flavour, and the other one decays to a *b* quark and a *W*, which subsequently decays to a charged lepton and a neutrino. This is illustrated in Fig. 2.

The relevant signal and SM background processes are simulated for LHC Run1-like conditions: proton–proton collisions at 8 TeV centre-of-mass energy, for an integrated luminosity of about 20 fb$^{-1}$. The detector simulation is based on Delphes, with parameters tuned on the CMS detector reconstruction and performances, but it does not include pileup. The analysis setup is validated by emulating an existing CMS search for rare top decays to *Zq* in $t\bar{t}$ events, showing reasonable results.

We find that a dedicated search by a single experiment using 20 fb$^{-1}$ of 8 TeV data could be sensitive to

$$\begin{aligned} BR(t \rightarrow e \bar{\mu } + ~\mathrm{jet}) \sim 6.3\times 10^{-5}, \end{aligned}$$

and we extrapolate that a sensitivity of ${\sim } 1.2 \times 10^{-5}$ (${\sim } 2 \times 10^{-6}$) could be reached with 100 fb$^{-1}$ at 13 TeV (3000 fb$^{-1}$ at 14 TeV). From Eq. (4), we see that the 100 fb$^{-1}$ data could impose $|\epsilon | \le 0.06$ for the $V \pm A$ operators, and $|\epsilon | \le 0.1$ for the $S \pm P$ and *LQ* operators. This analysis shows that the existing LHC data from Run1 can still be used to obtain interesting constraints on lepton-flavour violation. Although this is understandably not the priority focus in the times of the Run2 startup, let’s not to leave unchecked this possible path to New Physics.

## Appendix A: Operators

Consider the *S*-matrix element mediating $t\rightarrow q \ell _i^+ \ell _j^-$, where $\ell _k \in \{ e,\mu , \tau \}$, and suppose it is induced by local operators with momentum-independent coefficients, such that they can be added to the Standard Model Lagrangian. These operators should respect the $SU(3)\times SU(2)\times U(1)$ gauge symmetry of the Standard Model. However, since the New Physics scale that we explore is not much larger than the electroweak scale $m_t$, we should include dimension-eight operators constructed from two Higgs fields and 4 fermions, or two gradients and 4 fermions. Instead, we choose to work with $SU(3)\times U(1)$-invariant, but *not**SU*(2)-invariant, operators of dimension six. This is because a dimension-eight *SU*(2)-invariant operator, can be a dimension-six $SU(3)\times U(1)$-invariant operator:

8 $$\begin{aligned}&\frac{1}{\Lambda ^4} (\overline{E}_e \gamma _\alpha E_\mu ) ([\overline{Q}_2 H^c ] \gamma ^\alpha [H^T i\tau _2 Q_3 ])\nonumber \\&\quad \rightarrow \frac{v^2}{\Lambda ^4} (\overline{e} \gamma _\alpha P_R \mu ) (\overline{c} \gamma ^\alpha P_L t), \end{aligned}$$

where $Q_i$ is a quark doublet of *i*th generation, $E_j$ is a lepton singlet, $H^T= (H^+,H_0)$, $H^c = i\tau _2 H^*$ and SU(2) contraction is in square brackets.

This choice of *SU*(2)-non-invariant operators means that the coefficients of $$(\overline{e} \gamma _\alpha P_X \mu ) (\overline{s} \gamma ^\alpha P_L b) $$ and $$(\overline{e} \gamma _\alpha P_X \mu ) \times $$$$ (\overline{c} \gamma ^\alpha P_L t)$$ are taken as independent, and in particular, bounds on the first do not apply directly to the second. However, dressing the top operator with a *W* loop will generate the light quark vertex, which gives unavoidable bounds that are estimated in Appendix C. (Treating the *W* as dynamical while the New Physics is a contact interaction is unlikely to be a good approximation, but is the only way we can estimate whether the top operators are in tension with other observables.) In the case of $$\mu \rightarrow e \gamma $$, two or three loops are required to transform the top operators into the dipole operator, so we need an SU(2)-invariant formulation of the top operators. These dimension-six and -eight operators are given in Appendix A.2.

### A.1 $SU(3) \times U(1)$ invariant “top operators”

We are interested in contact interactions involving 2 charged leptons of different flavour, a top and a *c* or *u* quark. Such colour-singlet, electric charge-conserving, dimension-six operators will be referred to as “top operators”. The $V\pm A$ operators are:

9 $$\begin{aligned} \mathcal{O}^{LL}= & {} (\overline{e}_i \gamma ^\alpha P_L e_j ) (\overline{u}_q \gamma _\alpha P_L t ),\end{aligned}$$

10 $$\begin{aligned} \mathcal{O}^{LR}= & {} (\overline{e}_i \gamma ^\alpha P_L e_j ) (\overline{u}_q \gamma _\alpha P_R t ),\end{aligned}$$

11 $$\begin{aligned} \mathcal{O}^{RL}= & {} (\overline{e}_i \gamma ^\alpha P_R e_j ) (\overline{u}_q \gamma _\alpha P_L t ),\end{aligned}$$

12 $$\begin{aligned} \mathcal{O}^{RR}= & {} (\overline{e}_i \gamma ^\alpha P_R e_j ) (\overline{u}_q \gamma _\alpha P_R t ), \end{aligned}$$

and a redundant list of scalar/tensor operators is

13 $$\begin{aligned} \mathcal{O}^{S+ P,R}= & {} (\overline{e}_i P_R e_j ) (\overline{u}_qP_R t ) ,\end{aligned}$$

14 $$\begin{aligned} \mathcal{O}^{S+ P,L}= & {} (\overline{e}_i P_L e_j ) (\overline{u}_qP_L t ) ,\end{aligned}$$

15 $$\begin{aligned} \mathcal{O}^{S- P,R}= & {} (\overline{e}_i P_L e_j ) (\overline{u}_qP_R t ), \end{aligned}$$

16 $$\begin{aligned} \mathcal{O}^{S- P,L}= & {} (\overline{e}_i P_R e_j ) (\overline{u}_qP_L t ) ,\end{aligned}$$

17 $$\begin{aligned} \mathcal{O}^{T,R}= & {} (\overline{u}_q \sigma ^{\mu \nu } P_R e_j ) (\overline{e}_i \sigma _{\mu \nu } P_R t ),\end{aligned}$$

18 $$\begin{aligned} \mathcal{O}^{T,L}= & {} (\overline{u}_q \sigma ^{\mu \nu } P_L e_j ) (\overline{e}_i \sigma _{\mu \nu } P_L t ), \end{aligned}$$

19 $$\begin{aligned} \mathcal{O}^{LQ,R}= & {} (\overline{u}_q P_R e_j ) (\overline{e}_iP_R t ) =-\frac{1}{2}\mathcal{O}^{S+ P,R} + \frac{1}{8}\mathcal{O}^{T,R}, \nonumber \\ \end{aligned}$$

20 $$\begin{aligned} \mathcal{O}^{LQ,L}= & {} (\overline{u}_q P_L e_j ) (\overline{e}_i P_L t ) =-\frac{1}{2}\mathcal{O}^{S+ P,L} + \frac{1}{8}\mathcal{O}^{T,L} \end{aligned}$$

where *e*, *u* are Dirac spinors, *i*, *j* are unequal lepton-flavour indices, $q \in \{u,c\}$, and the last chiral superscript of the operators gives the chirality of the top.

The scalar operators $\mathcal{O}^{LQ,X}$ can be exchanged for the tensor operators $\mathcal{O}^{T,X}$, as shown in Eqs. (19) and (20). We will use the *LQ* and *T* operators interchangeably, because the $\mathcal{O}^{LQ,X}$ are more convenient in top decay, and the tensors at low energy. The tensors are used in the basis of [127]. Notice that $S+P$ operators are defined to have the same chiral projector twice, whereas for $S-P$ they are different. Since the last quark flavour label is fixed to *t*, $S + P$, $S-P$ and *LQ* operators appear twice, for both chiralities $t_L$ and $t_R$ (were we using arbitrary quark flavour indices *q*, *v*, then $[\mathcal{O}^{S+ P,R}_{ijqv}]^\dagger = \mathcal{O}^{S+ P,L}_{jivq}$). Finally, the scalar operator contracting quark to lepton spinors is given the name *LQ*; there are no such operators of the form $(\overline{u}_q P_R e_j ) (\overline{e}_j P_L u_t )$, because this is Fierz-equivalent to $\mathcal{O}^{RL}$.

A similar list can be constructed for down-type quarks. These will be relevant, because the top operators can generate the down operators at one loop. To reduce index confusion, down-type quarks will have Greek flavour indices $\alpha ,\beta \in \{d,s,b\}.$ There are also charge-current (CC) scalar operators

21 $$\begin{aligned}&\mathcal{O}^{CC} = (\overline{\ell }_i P_L \nu _j)(\overline{u}_q P_L d_\beta ),\nonumber \\&\quad \mathcal{O}^{CC,LQ} = (\overline{u}_q P_L \nu _j)(\overline{\ell }_i P_L d_\beta ) \end{aligned}$$

which will be relevant in setting bounds.

These operators are included in the Lagrangian with a dimensionful coefficient

22 $$\begin{aligned} -\frac{ 1}{\Lambda ^2} \equiv -2 \sqrt{2} G_F \epsilon . \end{aligned}$$

The $\epsilon$s have subscripts that identify the operator, and lepton-quark flavour superscripts: $2 \sqrt{2} G_F \epsilon _{LL}^{ijqt}$ would be the coefficient of $(\overline{e}_i \gamma ^\alpha P_L e_j ) (\overline{u}_q \gamma _\alpha P_L t )$, and $\epsilon _{LQ,X}^{ijqt}$ would be the coefficient of $(\overline{u}_q P_X e_j ) (\overline{e}_i P_X t )$ (so in particular, the index order is always $\bar{\ell } \ell \bar{q} q$, even for $\mathcal{O}^{LQ}$).

### A.2 $SU(3) \times SU(2) \times U(1)$ operators

Our $V\pm A$ operators can be identified as components of SU(2) invariant dimension-six operators:

23 $$\begin{aligned}&\mathcal{O}^{LL} \subset \mathcal{O}_{L Q}^{(1)}= (\overline{L}_e \gamma ^\alpha L_\mu ) (\overline{Q}_q \gamma _\alpha Q_t)\end{aligned}$$

24 $$\begin{aligned}&\quad =[(\overline{e} \gamma ^\alpha P_L \mu ) + (\overline{\nu }_e \gamma ^\alpha P_L \nu _\mu )][(\overline{u}_q \gamma _\alpha \tau ^a P_L t ) + (\overline{d}_q \gamma _\alpha \tau ^a P_L b )],\nonumber \\&\mathcal{O}^{LR} \subset \mathcal{O}_{LU}= (\overline{L}_e \gamma ^\alpha L_\mu ) (\overline{U}_q \gamma _\alpha U_t ), \end{aligned}$$

25 $$\begin{aligned}&\mathcal{O}^{RL} \subset \mathcal{O}_{EQ}=(\overline{E}_e \gamma ^\alpha E_\mu ) (\overline{Q}_q \gamma _\alpha Q_t ), \end{aligned}$$

26 $$\begin{aligned}&\mathcal{O}^{RR} =\mathcal{O}_{EU}=(\overline{E}_e \gamma ^\alpha E_\mu ) (\overline{U}_q \gamma _\alpha U_t ) \end{aligned}$$

where *L*, *Q* are doublets and *E*, *U* are singlets *t* is a third generation quark index, *q* a first or second generation quark index, and we explicitly choose lepton indices $i =e$ and $j = \mu$. In the SU(2) invariant list, there is an additional operator at dimension six, which generates $\mathcal{O}_{L L}$ in combination with charged-current interactions:

27 $$\begin{aligned} \mathcal{O}_{L Q}^{(3)}= & {} (\overline{L}_i \gamma ^\alpha \tau ^aL_j ) (\overline{Q}_q \gamma _\alpha \tau ^a Q_t ) \nonumber \\= & {} 2 (\overline{\nu }_i \gamma ^\alpha P_L e_j ) (\overline{d}_q \gamma _\alpha P_L t ) + 2 (\overline{e}_i \gamma ^\alpha P_L \nu _j ) (\overline{u}_q \gamma _\alpha P_L b ) \nonumber \\&+ [(\overline{\nu }_i \gamma ^\alpha P_L \nu _j ) - (\overline{e}_i \gamma ^\alpha P_L e_j )] [(\overline{u}_q \gamma _\alpha P_L t )\nonumber \\&-(\overline{d}_q \gamma _\alpha P_L b )]. \end{aligned}$$

Alternatively, at dimension eight, SU(2) invariant operators can be constructed to contain only an up-type quark current, by contracting quark and Higgs doublets. An example is given in Eq. (8).

Then there are two $S + P$ operators:

$$\begin{aligned} \mathcal{O}^{S+ P,R}= & {} (\overline{e} P_R \mu ) (\overline{u}_qP_R t ) \subset \mathcal{O}_{L_1 E_2 Q_2 U_3} \\ \mathcal{O}^{S+ P,L}= & {} (\overline{e} P_L \mu ) (\overline{u}_qP_L t ) \subset \mathcal{O}_{L_2E_1 Q_3 U_2}^\dagger , \end{aligned}$$

which are components of the same SU(2) invariant operator:

28 $$\begin{aligned} \mathcal{O}_{LE Q U}= & {} (\overline{L}_i^A E_j )\epsilon _{AB} (\overline{Q}^B_q U_t )\nonumber \\= & {} -(\overline{\nu }_i e_j ) (\overline{d}_q u_t ) + (\overline{e}_i P_R e_j ) (\overline{u}_qP_R u_t) \end{aligned}$$

and there are the LQ or tensor operators, which are components of the dimension-six, SU(2)-invariant operator

29 $$\begin{aligned} \mathcal{O}_{LUQE}= & {} (\overline{L}_i^A U_t ) \epsilon _{AB} (\overline{Q}^B_q E_j ) =-\frac{1}{2} \mathcal{O}_{LE Q U}\nonumber \\&+\frac{1}{8} (\overline{L}_i^A \sigma ^{\mu \nu } E_j ) \epsilon _{AB} (\overline{Q}^B_q \sigma _{\mu \nu } U_t), \end{aligned}$$

and finally there are two $S-P$ operators, which can be identified with an SU(2)-invariant operator of dimension eight:

30 $$\begin{aligned} v^2 \mathcal{O}^{S- P,R}= & {} v^2 (\overline{e} P_L \mu ) (\overline{u}_qP_R t )\nonumber \\= & {} (\overline{E}_1 [H^\dagger L_2] ) ([\overline{Q}_q H^c ] U_3)\end{aligned}$$

31 $$\begin{aligned} v^2 \mathcal{O}^{S- P,L}= & {} ([\overline{L}_e H ] E_\mu ) (\overline{U}_q [H^T i\tau _2 Q_t] ) \end{aligned}$$

where, for instance, $[H^\dagger L_2] = v \mu _L$. Notice that our $S+P$ and tensor operators can also be obtained at dimension eight without associated charged-current interactions:

32 $$\begin{aligned} (\overline{L}_i H E_j ) (\overline{Q}_q H^c U_t ) = v^2 (\overline{e}_i P_R e_j ) (\overline{u}_qP_R u_t ). \end{aligned}$$

## Appendix B: top operators in $\mu \rightarrow e \gamma$

In this appendix, we wish to close the quark lines of a top operator, and attach a photon (and a Higgs vev) to the resulting diagram, such that it contributes to $\mu \rightarrow e \gamma$. These two and three loop estimates rely on using SU(2)-invariant operators (of dimension six or eight), because using the $d_L$ and $\nu _L$ components of doublets will allow one to avoid GIM suppression. Then we estimate the contribution to $\mu \rightarrow e \gamma$ by power counting selected diagrams.

### B.1 Parametrising $\mu \rightarrow e \gamma$

The decay $\mu \rightarrow e \gamma$ is mediated by the dipole operator, which can be added to the Standard Model Lagrangian as [1–3]

33 $$\begin{aligned} \mathcal{L}_{meg}= & {} -\frac{4G_F}{\sqrt{2}} m_\mu ( A_R \overline{\mu _R} \sigma ^{\alpha \beta } e_L F_{\alpha \beta } + A_L \overline{\mu _L} \sigma ^{\alpha \beta } e_R F_{\alpha \beta })\nonumber \\= & {} -\frac{4G_F}{\sqrt{2}} y_\mu ( A_R \overline{\mu _R} \sigma ^{\alpha \beta } [H^\dagger L_e] F_{\alpha \beta }\nonumber \\&+A_L [\overline{L_\mu } H] \sigma ^{\alpha \beta } e_R F_{\alpha \beta }) \end{aligned}$$

where, in the second formulation, $y_\mu$ is the muon Yukawa coupling such that $y_\mu v= m_\mu$, and the fermion part has been written in SU(2)-invariant form, to emphasise that the dipole operator is of dimension six, and has a Higgs leg. This operator gives a branching ratio:

34 $$\begin{aligned} BR(\mu \rightarrow e \gamma )= 384 \pi ^2 (|A_R|^2 + |A_L|^2) <5.7\times 10^{-13}\nonumber \\ \end{aligned}$$

where we quote the upper bound from [4]. If $|A_R| = |A_L|$, this implies $|A_X| <8.6\times 10^{-9}$. However, in the case of our diagrams, there will be a sum over three colours at the amplitude-squared level, so its convenient to divide by $\sqrt{3}$, and impose

35 $$\begin{aligned} |A_X| <5.0 \times 10^{-9}. \end{aligned}$$

This is a small number. We can estimate the contribution of top operators to be suppressed by various factors:

1. two loops, and in some cases three. Two loops are necessary to close the quark legs, because they are of different flavour, so must interact with a *W*.
2. CKM, because the quark legs are of different generation. This is ${\sim } .04$ for $c\leftrightarrow t$ flavour change, ${\sim } .008$ for $u \leftrightarrow t$ flavour change.
3. GIM: if the W is exchanged between two up-type quark lines (see Fig. 5), then the diagram is further suppressed by a GIM factor ${\sim } m_b^2/m_W^2 \simeq 1/400$ (for $m_b=4$ GeV). This can be avoided by exchanging the *W* between the quark and lepton lines.
4. mass insertions: may be required on the quark line to transform singlet into doublet quarks. However, the dipole operator of Eq. (33) is defined with a Higgs leg attached to the muon line; so this can be an amplification factor if the Higgs can be attached to a heavier fermion.

So for our $SU(3)\times U(1)$ invariant top operators with coefficient $\epsilon \frac{4G_F}{\sqrt{2}}$, one could anticipate

36 $$\begin{aligned}&A_X \sim \frac{ e g^2/2}{(16\pi ^2)^2} \epsilon \times \hbox {CKM} \times \hbox {GIM} \times \hbox {m}\text {-}\hbox {insertations}\nonumber \\&\quad \simeq \left\{ \begin{array}{ll} 10^{-7}&{}\epsilon \times \hbox {GIM} \times \hbox {m}\text {-}\hbox {insertations} , q = c\\ 2\times 10^{-8} &{}\epsilon \times \hbox {GIM} \times \hbox {m}\text {-}\hbox {insertations}, q = u \end{array} \right. \end{aligned}$$

where, in some diagrams, the GIM factor is absent, and/or the mass insertion factor can be ${\gg } 1$. Comparing to Eq. (35) suggests that $\mu \rightarrow e \gamma$ can provide relevant constraints on the $\epsilon$s, unless there is more suppression than CKM and two loops.

### B.2 Estimated contributions to $\mu \rightarrow e \gamma$

It is convenient to pretend that the New Physics scale is ${\gg } v$, which allows one to study whether (the SU(2) invariant generalisations of) the top operators mix to the dipole in electroweak running down to *v*. The aim is to add electroweak boson loops to the top operator such that the quark lines can be closed, and the dipole operator of Eq. (33) is obtained. Closing the quark lines requires changing the quark flavours. The lepton spinor contraction of the dipole is tensorial, so for all non-tensor top operators, it must be changed to this form by involving a lepton line in a loop.

1. Consider first the SU(2) invariant versions of the $V\pm A$ operators of Eqs. (23)–(26). These operators have no $(\bar{e} \gamma \nu )(\bar{u} \gamma d)$ components, so two *W* vertices are required on the quark loop (see the first diagram of Fig. 5). This gives 2-loop and CKM suppression. For singlet quark currents, also mass insertions are required on the *t* and *q* lines, and the diagram will be suppressed by a GIM factor ${\sim } m_b^2/m_W^2$ (these mass factors correspond to the crosses on the quark loop of the first diagram of Fig. 5). To modify the spinor contraction between the leptons, a $\gamma ,Z$ could be exchanged between the quark loop and an external lepton line, which gives an additional suppression ${\sim } e^2/(16\pi ^2)$. So for singlet quark currents (the operators of Eqs. (24) and (26)):
  37 $$\begin{aligned} A_X \sim \frac{ e^3 g^2/2}{(16\pi ^2)^3} \epsilon \times V_{qb} \frac{m_b^2}{m_W^2} \frac{m_q}{m_t} \simeq 10^{-15} \epsilon \times \frac{V_{qb}}{.04}, \end{aligned}$$
  and for doublet quark currents, where the GIM and $m_q$ suppression can be avoided by emitting *d*-type quarks from the operator:
  38 $$\begin{aligned} A_X \sim \frac{ e^3 g^2/2}{(16\pi ^2)^3} \epsilon \times V_{tq} \simeq 10^{-10} \epsilon \times \frac{ V_{tq} }{.04}. \end{aligned}$$
  These loops give no bound on $\epsilon _{LL}$, $\epsilon _{LR}$, $\epsilon _{RL}$ or $\epsilon _{RR}$.
2. However, if the $\mathcal{O}_{LL}$ operator is generated as a component of the triplet SU(2)-invariant operator of Eq. (27), then there is a charged current contact interaction, which allows for *W* exchange between the lepton and quark lines and avoids any GIM suppression. The diagram is similar to Fig. 5 on the right, but with $t_L$ and $e_L$ instead of $t_R$ and $e_R$, and a mass insertion on the external $\mu$ rather than the *t*. An estimate gives
  39 $$\begin{aligned} A_X \sim \frac{ e g^2/2}{(16\pi ^2)^2} \epsilon \times \hbox {CKM} \simeq 10^{-7} \epsilon \times \frac{\mathrm{CKM}}{.04}, \end{aligned}$$
  which imposes $\epsilon _{LL} \lesssim 0.03$ for $q=c$, or $\epsilon _{LL} \lesssim 0.5$ for $q=u$. Note that the B decay constraints, which are estimated below more rigorously than these ones, are tighter.
3. Consider now the $S+P$ or *T* operators which arise at dimension six, which can induce the second diagram of Fig. 5. The grey blob represents the charge-changing component of the operator, involving $t_R$ and either $d_L$ or $s_L$. The contribution to $\mu \rightarrow e \gamma$ is two-loop and CKM suppressed, but enhanced by $m_t/m_\mu$, because the chirality flip for the leptons is in the contact interaction, so the Higgs leg of the dipole operator can attach to the mass insertion required to flip the chirality of the top. This gives
  40 $$\begin{aligned} A_X \sim \frac{ e g^2/2}{(16\pi ^2)^2} \epsilon \times \left\{ \begin{array}{c} V_{ts} \\ V_{td} \end{array} \right\} \times \frac{m_t}{m_\mu } \simeq \left\{ \begin{array}{rl} 2 \times 10^{-4} &{}\epsilon ^{ct} \\ 6 \times 10^{-5} &{}\epsilon ^{ut} \end{array} \right. \nonumber \\ \end{aligned}$$
  which implies $\epsilon _{S+P,R}, \epsilon _{T,R} \lesssim 10^{-4}\rightarrow 10^{-5}$! In the case where the third generation quark is a doublet, there would be a $b_L$ and a charm or up mass insertion, so that
  41 $$\begin{aligned} A_X \sim \frac{ e g^2/2}{(16\pi ^2)^2} \epsilon \times V_{qb} \frac{m_q}{m_\mu } \sim \left\{ \begin{array}{rl} 10^{-6} &{} \epsilon ^{ct} \\ 6 \times 10^{-10}&{} \epsilon ^{ut} \end{array}, \right. \end{aligned}$$
  which would suggest $\epsilon _{S+P,R}^{ct}, \epsilon _{T,R}^{ct} \lesssim 10^{-3}$.
4. Finally, consider an $S\pm P$ or *T* operator of dimension eight, so with two Higgs legs. If the Higgs are both neutral vevs, then the contact interaction involves up-type quarks, and gives diagrams whose contribution to $\mu \rightarrow e \gamma$ is suppressed by three loops, CKM and GIM. Instead, focus on diagrams where the Higgs legs are charged, so the 4-fermion interaction is charge-changing for the leptons and quarks, and the Higgs loop has to close (two external Higgs legs can be attached to the top line, to keep the operator at dimension eight, which gives negligible suppression because $y_t \sim 1$). Due to the Higgs loop, the estimate for $A_X$ will be suppressed by ${\sim }10^{-2}$ with respect to the previous discussion of $S+P$ and *T* at dimension six. Since we do not know whether our “top operators” correspond to SU(2) invariant operators of dimension six or eight, we conservatively retain the dimension-eight bounds, which imply that for $S\pm P$ and *T* operators involving a singlet $t_R$
  42 $$\begin{aligned} \epsilon _{T,R}^{ct},\quad \epsilon _{S\pm P,R}^{ct}\lesssim 10^{-3},\qquad \epsilon _{T,R}^{ut},\quad \epsilon _{S\pm P,R}^{ut} \lesssim 10^{-2}.\nonumber \\ \end{aligned}$$

Similar estimates can be made for the contributions of top operators to $\tau \rightarrow \ell \gamma$. The current experimental upper bounds [128, 129] are $\Gamma (\tau \rightarrow e \gamma ) < 1.8\times 10^{-7} \Gamma (\tau \rightarrow e\nu \bar{\nu })$ and $\Gamma (\tau \rightarrow \mu \gamma ) < 2.5 \times 10^{-7} \Gamma (\tau \rightarrow \mu \nu \bar{\nu })$, which imply, with the assumptions leading to Eq. (35), that $A_X(\tau \rightarrow \ell \gamma ) \lesssim 3\times 10^{-6}$. For dimension-eight, $S\pm P$ or tensor operators, this would give $\epsilon ^{\ell \tau ut} \lesssim 100$ and $\epsilon ^{\ell \tau ct} \lesssim 10$.

## Appendix C: top operators in meson decays

Dressing a top operator with a *W* loop, where the *W* attaches to the top leg, generates a contact interaction among light fermions, where the *t* is replaced by *b*, *s* or *d*. So the first step in obtaining bounds on the top operators is to collect up-to-date bounds on the relevant 2-quark–2-lepton operators involving *d*-type quarks. These are listed in Tables 6, 7 and 8, updated from [103]. In Sect. C.2, we estimate the loop factors, which, when multiplied by the top-operator coefficients, give the coefficients of light-fermion operators that are constrained by Tables 6, 7 and 8. Finally, in Sect. C.3, we combine the loop factors and tables to obtain the bounds on top-operator coefficients given in Tables 1 and 2, and briefly discuss possible cancellations among the operators.

### C.1 Updated rare decay bounds

**Table 6:** Constraints on the dimensionless coefficient $\epsilon ^{ij\alpha \beta }$ of 4-fermion interactions $2 \sqrt{2} G_F (\bar{e}_{i} \gamma ^{\mu } P_{L,R} e_{j})(\bar{d}_{\alpha }\gamma _{\mu } P_{L,R} d_{\beta })$. $P_{L,R}$ can be $P_{L}$ or $P_{R}$. The generation indices $ij\alpha \beta$ are given in the first column, and the best bound in column 3. It arises from the observable of column 4, and the experimental value we used is given in column 5. In column 2 is given the linear combination of $\epsilon$s to which the bound applies. All bounds apply under permutation of the lepton and/or quark indices

$ij\alpha \beta$		Constraint on $\epsilon ^{ij \alpha \beta }$	Observable	Experimental value
$e\mu ds$	$(\epsilon _{LL} - \epsilon _{LR}),~ (\epsilon _{RR} -\epsilon _{RL})$	$3.0\times 10^{-7}$	$BR(K^{0}_{L}\rightarrow \bar{e}\mu )$	${<}4.7\times 10^{-12}$
$e\mu db$	$(\epsilon _{LL} - \epsilon _{LR}),~ (\epsilon _{RR} -\epsilon _{RL})$	$3.0\times 10^{-4}$	$BR(B^{0}\rightarrow \bar{e}\mu )$	${<}2.8\times 10^{-9}$
	$\epsilon _{LL} +\epsilon _{LR} ,~ \epsilon _{RR} +\epsilon _{RL}$	$1.3\times 10^{-4}$	$\frac{BR(B^{+}\rightarrow \pi ^+ \bar{e}\mu )}{BR(B^{0}\rightarrow \pi ^- \bar{e}\nu )}$	${<}1.3\times 10^{-3}$
$e\mu sb$	$\epsilon _{LL} +\epsilon _{LR},~ \epsilon _{RR} +\epsilon _{RL}$	$1.0 \times 10^{-4}$	$\frac{BR(B^{+}\rightarrow K^{+}\bar{e}\mu )}{{BR(B^{+}\rightarrow D^{0}\bar{e}\nu _{e})}}$	${<}\frac{9.1\times 10^{-8}}{2.2\times 10^{-2}}$
$e\tau ds$	$(\epsilon _{LL} - \epsilon _{LR}),~ (\epsilon _{RR} -\epsilon _{RL})$	$4.1\times 10^{-4}$	$\frac{BR( \tau \rightarrow {e} K)}{BR( \tau \rightarrow \bar{\nu } K)}$	${<}\frac{2.6 \times 10^{-8}}{7.0 \times 10^{-3}}$
$e\tau db$	$(\epsilon _{LL} - \epsilon _{LR}),~ (\epsilon _{RR} -\epsilon _{RL})$	$2.1\times 10^{-3}$	$BR(B^{0}\rightarrow \bar{e}\tau )$	${<}2.8\times 10^{-5}$
$e\tau sb$	$\epsilon _{LL} +\epsilon _{LR},~ \epsilon _{RR} +\epsilon _{RL}$	$2.0 \times 10^{-3}$	$\frac{BR(B^+ \rightarrow K^+ \bar{\tau } e)}{{BR(B^{+}\rightarrow D^{0}\bar{\tau }\nu )}}$	${<}\frac{3.0\times 10^{-5}}{7.7\times 10^{-3}}$
$\mu \tau ds$	$(\epsilon _{LL} - \epsilon _{LR}),~ (\epsilon _{RR} -\epsilon _{RL})$	$4.3\times 10^{-4}$	$\frac{BR( \tau \rightarrow {\mu } K)}{BR( \tau \rightarrow \bar{\nu } K)}$	${<} \frac{2.3 \times 10^{-8}}{7.0 \times 10^{-3}}$
$\mu \tau db$	$(\epsilon _{LL} - \epsilon _{LR}),~ (\epsilon _{RR} -\epsilon _{RL})$	$1.6\times 10^{-3}$	$BR(B^{0}\rightarrow \bar{\mu }\tau )$	${<}2.2\times 10^{-5}$
$\mu \tau sb$	$\epsilon _{LL} +\epsilon _{LR},~ \epsilon _{RR} +\epsilon _{RL}$	$3.1 \times 10^{-3 }$	$\frac{BR(B^+ \rightarrow K^+ \bar{\tau } \mu )}{{BR(B^{+}\rightarrow D^{0}\bar{\tau }\nu )}}$	${<}\frac{4.8\times 10^{-5}}{7.7\times 10^{-3}}$

The bounds from the decay rate of a pseudoscalar meson *M*, are obtained using the usual formula:

43 $$\begin{aligned} \Gamma (M \rightarrow l^{i}\bar{l}^{j})= & {} \frac{kG_F^2}{\pi m^{2}_{M}} \left\{ \widetilde{P}^{2} [(|\epsilon _{S \pm P,R} - \epsilon _{S\pm P,L}|^{2})\right. \nonumber \\&\times (m^{2}_{M}-m^{2}_{i}-m^{2}_{j}) + 4 \epsilon _{L} \epsilon _{R} m_1 m_2]\nonumber \\&+(|\epsilon _{XR} - \epsilon _{XL}|^{2}) \widetilde{A}^{2}[(m^{2}_{M}-m^{2}_{i} -m^{2}_{j})\nonumber \\&\times \left. (m^{2}_{i}+m^{2}_{j}) +4m^{2}_{i}m^{2}_{j}]\right\} , \end{aligned}$$

where scalar–vector interference was neglected. In current algebra, the quark currents are

44 $$\begin{aligned}&\widetilde{A}P^\mu = \frac{1}{2} \langle 0| \overline{q} \gamma ^\mu \gamma ^5 q |M \rangle = \frac{f_{M}P^\mu }{2}\nonumber \\&\quad \widetilde{P}= \frac{1}{2} \langle 0| \overline{q}_o \gamma ^5 q_n |M \rangle = \frac{f_{M}m_{M}}{2}\frac{m_{M}}{m_{o}+m_{n}}, \end{aligned}$$

where *o*, *n* are flavour indices, and *k* is the magnitude of the final state 3-momenta in the centre-of-mass frame:

45 $$\begin{aligned} k^{2}=\frac{1}{4m^{2}_{M}}[(m^{2}_{M}-(m_{i}+m_{j})^{2}) (m^{2}_{M}-(m_{i}-m_{j})^{2})].\nonumber \\ \end{aligned}$$

The decay $B^+\rightarrow K^+ e^-\ell ^+$ can be used to constrain $\epsilon ^{e \ell sb}$, with a suitable approximation for the hadronic matrix element. For this, we follow the Babar exclusive determination of $V_{cb}$ [130] from $B^+\rightarrow D^0 \ell ^+\nu$, where the differential decay rate is parametrised with a kinematic term depending on $q^2= (p_B - p_D)^2$ and a form factor that is a function of the inner product $\omega$ of *B* and *D* 4-velocities:

$$\begin{aligned} \frac{\mathrm{d} \Gamma }{\mathrm{d} \omega }= & {} \frac{G_F^2|V_{cb}|^2m_B^3}{8 \times 48\pi ^3}(m_B+m_D)^2 (1 + \mathcal{O}(m_D^2/m_B^2)\\&+\cdots )\mathcal{G}^2(\omega ) \end{aligned}$$

where $\cdots$ includes the $q^2$ terms. To extract a bound on $\epsilon$ from $B^+\rightarrow K^+ e^-\tau ^+$, we suppose the form factors are similar and impose

46 $$\begin{aligned} \frac{\Gamma (B^+\rightarrow K^+ e^-\tau ^+)}{\Gamma (B^+\rightarrow D^0 \ell ^+\nu )} = \frac{\epsilon ^2}{|V_{cb}|^2} \frac{(m_B+m_K)^2}{(m_B+m_D)^2}, \end{aligned}$$

which is slightly weaker than the bound in [103], for the case $\ell = \mu$.

For $S\pm P$ operators, we included $\tau \rightarrow K_S \ell$ bounds that were not in [103], but that were used in [131].

### C.2 Estimating the loops

Consider first the $$V \pm A$$ operators $$\mathcal{O}^{YR}$$, involving the singlet $$t_R$$, with coefficient $$ 2\sqrt{2} G_F \epsilon _{YR}^{ijqt}$$, $$q \in \{ c,u \}$$. Dressing with a *W* loop as in Fig. 1 gives the operators $$ \mathcal{O}^{YL}_{ij \alpha \beta } =$$$$(\overline{\ell }_i \gamma _\alpha P_Y \ell _j)$$$$(\overline{d}_\alpha \gamma ^\alpha P_L d_\beta )$$ with coefficients

47 $$\begin{aligned} \epsilon _{YL}^{ij \alpha \beta }\simeq & {} -\frac{g^2 m_t m_{q} V_{t\beta } V^*_{q \alpha }}{16\pi ^2 (m_t^2 - m_W^2) } \log \left( \frac{m_t^2}{m_W^2} \right) \epsilon _{YR}^{ijqt}\nonumber \\\simeq & {} -\frac{2\alpha _{em}}{3} V_{t\beta } V^*_{q\alpha } \frac{m_{q}}{m_t} \epsilon _{YR}^{ijqt} \end{aligned}$$

where $(\beta ,\alpha ) \in \{ (b,s), (b,d),(s,d) \}$, $q \in \{ c,u \}$. The log is ${\simeq } 1.54 \simeq \pi /2$. The numerical factor arising from the loop depends on the light external quark indices $\beta ,\alpha$, as well as the quark flavour *q* participating with the top in the contact interaction. It is given in Table 9. It is clear that the loop gives a significant suppression, due to the CKM elements and light quark masses.

The $$V \pm A$$ operators $$\mathcal{O}^{YL}$$, which involve doublet top quarks, have a larger mixing to down-type quark operators. Dressing with a *W* loop as in Fig. 1 gives the operator $$ \mathcal{O}^{YL}_{ij\alpha \beta }=(\overline{\ell }_i \gamma _\alpha P_Y \ell _j)$$$$(\overline{d}_\alpha \gamma ^\alpha P_L d_\beta )$$, with a log divergent coefficient, unsuppressed by light quark masses:

48 $$\begin{aligned} \epsilon _{YL}^{ij\alpha \beta }\simeq & {} \frac{g^2 V_{t\beta } V^*_{q\alpha }}{32\pi ^2 } \left( \frac{m_W^2}{m_t^2 - m_W^2} \log \left[ \frac{m_t^2}{m_W^2} \right] -\log \left[ \frac{\Lambda ^2}{m_t^2} \right] \right) \epsilon _{YL}^{ijqt}\nonumber \\\simeq & {} - \frac{3\alpha _{em}}{4\pi } V_{t\beta } V^*_{q\alpha } \epsilon _{YL}^{ijqt} \end{aligned}$$

where $$(\beta ,\alpha ) \in \{ (b,s), (b,d),(s,d) \}$$, $$q \in \{ c,u \}$$, only terms with logs were retained, and $$\Lambda \simeq 3 m_t$$ in the log to obtain the second approximation^4^ and the numerical factors in Table 9. The top operators $$\mathcal{O}_{ijqt}^{LL}$$ can also generate Charged Current (CC) operators $$(\overline{\nu }_i \gamma ^\rho P_L \ell _j)$$$$(\overline{u}_q \gamma ^\rho P_L d_\beta )$$, suppressed only by $$V_{t\beta }$$, however, the bounds on the coefficients of such CC operators are weaker than the bounds on FCNC operators, so the best limit on the top operators arises from the loop given in Eqs. (47) and (48).

**Table 7:** Constraints on $\epsilon _{S \pm P,X}$, for the $ij \alpha \beta$ index combination given in the first column, where the $\epsilon$s are coefficients of the operators $\mathcal{O}_{S \pm P,X}=(\bar{e}_{i}P_Y e_{j})(\bar{d}_{\alpha }P_Xd_{\beta })$, in a basis containing also tensor operators. The second column is the constraint, which arises from the observable given in column 3. The experimental value used is the last column. These bounds are also valid under lepton and/or quark index permutation

$ij\alpha \beta$	Constraint on $\epsilon ^{ij\alpha \beta }$	Observable	Experimental value
$\mu eds$	$9.0\times 10^{-9}$	$BR(\overline{K^{0}_{L}}\rightarrow \bar{\mu }e)$	${<}4.7\times 10^{-12}$
$e\mu bd$	$8.1\times 10^{-6}$	$BR(B^{0}\rightarrow \bar{e}\mu )$	${<}9.2\times 10^{-8}$
$e\mu bs$	$2.9\times 10^{-5}$	$BR(B^{0}_{s}\rightarrow \bar{e}\mu )$	${<}1.1\times 10^{-6}$
$e\tau ds$	$2.9\times 10^{-4}$	$BR(\tau \rightarrow K_S^0 e)$	${<}2.6\times 10^{-8}$
$e\tau db$	$5.6\times 10^{-4}$	$BR(\overline{B^{0}}\rightarrow \bar{e}\tau )$	${<}2.8\times 10^{-5}$
$$e\tau sb$$			
$\mu \tau bd$	$5.0\times 10^{-4}$	$BR(B^{0}\rightarrow \bar{\mu }\tau )$	${<}2.2\times 10^{-5}$
$\mu \tau ds$	$2.6\times 10^{-4}$	$BR(\tau \rightarrow K_S^0 e)$	${<}2.3\times 10^{-8}$
$$\mu \tau sb$$			

**Table 8:** Constraints from “charged-current” processes on $S \pm P$ operators. This bound applies to $\epsilon ^{ij\beta q}_{CC}$, in a basis using tensor operators. The first column is the index combination $ij\beta q$, the second is the constraints, which arise from the observable given in column 3. The experimental value used is the last column. $\nu _i$ is any flavour of neutrino

$(\bar{\nu }_{i}{e}_{j})(\bar{d}_{\beta }u_{q})$	Constraint on $\epsilon ^{ij\beta q}$	Observable	Experimental value
$\nu _{i}edu$	$1.6 \times 10^{-5}$	$R_{\pi }$	$(1.230\pm 0.004)\times 10^{-4}$
$\nu _{i}edc$	$1.4 \times 10^{-3}$	$\Gamma (D^+ \rightarrow \bar{e} \nu _i)$	${<}8.8 \times 10^{-6}$
$\nu _{i}e su$	$1.5\times 10^{-5}$	$BR(K^+ \rightarrow \bar{e} \nu _i)$	$(1.55\pm 0.07)\times 10^{-5}$
$\nu _{i}esc$	$3.9 \times 10^{-3}$	$BR(D_s^+ \rightarrow \bar{e} \nu _i)$	${<}8.3 \times 10^{-5}$
$\nu _{i}ebu$	$7.8\times 10^{-5}$	$BR(B^{+}\rightarrow \bar{e}\nu )$	${<}9.8\times 10^{-7}$
$$\nu _{i}ebc$$			
$\nu _{i}\mu du$	$3.2 \times 10^{-3}$	$R_{\pi }$	$(1.230\pm 0.004)\times 10^{-4}$
$\nu _{i}\mu dc$	$3.7 \times 10^{-3}$	$BR(D^{+}\rightarrow \bar{\mu }\nu )$	$(3.82\pm 0.33)\times 10^{-4}$
$\nu _{i}\mu su$	$3.0\times 10^{-3}$	$R_{K}$	$(2.44\pm 0.11)\times 10^{-5}$
$\nu _{i} \mu sc$	$1.5\times 10^{-2}$	${BR(D^{+}_{s}\rightarrow \bar{\mu }\nu )}$	$5.56\times 10^{-3}$
$\nu _{i}\mu bu$	$7.7\times 10^{-5}$	$BR(B^{+}\rightarrow \bar{\mu }\nu )$	${<}1.0\times 10^{-6}$
$$\nu _{i} \mu bc $$			
$\nu _{i} \tau du$	$8.0\times 10^{-2}$	$BR(\tau \rightarrow \pi ^+ \nu )$	$(1.083 \pm 0.006) \times 10^{-1}$
$\nu _{i} \tau dc$	$5.2\times 10^{-2}$	$BR(D^{+}\rightarrow \bar{\tau } \nu )$	${<}1.2 \times 10^{-3}$
$\nu _{i} \tau su$	$2.6\times 10^{-2}$	$BR(\tau \rightarrow K^+ \nu )$	$(7.0\pm 0.1) \times 10^{-3}$
$\nu _{i} \tau sc$	$4.9\times 10^{-2}$	$BR(D^{+}_{s}\rightarrow \bar{\tau }\nu )$	$5.54 \times 10^{-2}$
$\nu _{i} \tau bu$	$7.2\times 10^{-4}$	$BR(B^{+}\rightarrow \bar{\tau }\nu _{\tau })$	$(1.1 \pm 0.3) \times 10^{-4}$
$$\nu _{i} \tau bc $$			

The $$S \pm P$$ operators $$\mathcal{O}^{ S\pm P,X}_{ijqt}$$ involving either $$t_L$$ ($$X=L$$) or $$t_R$$ ($$X=R$$), will generate $$S \pm P$$ operators for down-type quarks $$ \mathcal{O}^{S\pm P,R}_{ij\alpha \beta } =$$$$(\overline{e}_i P_Y e_j)$$$$(\overline{d}_\alpha P_R d_\beta )$$, where it is always the heavier $$d_\beta $$ that is right-handed, because there is a mass insertion on an external quark leg. The lepton current is unaffected by the loop, so it will have the same chirality in the induced down-quark operator as it had in the top-LFV operator. There is also a mass insertion on the right-handed internal quark line, so the coefficient is $$\propto m_t$$ in the presence of $$\mathcal{O}^{ S\pm P,R}_{ijqt}$$:

49 $$\begin{aligned} \epsilon _{ S\pm P,R}^{ij \alpha \beta }\sim & {} \frac{g^2 m_t m_\beta V_{t\beta } V^*_{q\alpha }}{32\pi ^2 (m_t^2-m_W^2)} \left( 1 - \frac{m_W^2}{m_t^2 - m_W^2} \log \frac{m_t^2}{m_W^2}\right) \epsilon _{ S\pm P,R}^{ijqt}\nonumber \\\simeq & {} \frac{\alpha _{em}}{3\pi } V_{t\beta } V^*_{q\alpha }\frac{ m_\beta }{ m_t} \epsilon _{ S\pm P,R}^{ijqt}. \end{aligned}$$

The result in the presence of $$\mathcal{O}^{ LQ,R}_{ijqt}$$ is similar: the *LQ* operator for down-type quarks, $$ \mathcal{O}^{LQ,R}_{ij\alpha \beta }=(\overline{d}_\alpha P_R e_j)$$$$(\overline{e}_i P_R d_\beta )$$, is generated with a coefficient obtained by replacing $$\epsilon _{ S\pm P,R}^{ijqt} \rightarrow -\epsilon _{ LQ,R}^{ijqt}$$ in Eq. (49).

In the presence of $\mathcal{O}^{ S\pm P,L}_{ijqt}$ (or $\mathcal{O}^{ LQ,L}_{ijqt}$), the loop factor is $\propto m_q m_\beta$, which is negligibly small. So we obtain no interesting bounds on the $\mathcal{O}^{ S- P,L}_{ijqt}$ operators. However, the operators $\mathcal{O}^{S+P,L}_{ijqt}$ (and $\mathcal{O}^{LQ,L}_{ijqt}$) contain $t_L$ and $e_{Lj}$, between whom the *W* could be exchanged as indicated in the right diagram of Fig. 1. This dressing of the top operator $\mathcal{O}^{S+P,L}_{ijqt}$ will generate $\mathcal{O}^{CC}_{ijqt \beta }$ and a tensor operator, in the linear combination corresponding to $\mathcal{O}^{CC,LQ}$ of Eq. (21). The loop coefficient is unsuppressed by the masses, as in Eq. (48):

50 $$\begin{aligned} \epsilon _{CC,LQ}^{ij q\beta }\simeq & {} -\frac{ g^2 V_{t\beta } }{16 \pi ^2 } \left( \frac{m_W^2}{m_t^2 - m_W^2} \log \left[ \frac{m_t^2}{m_W^2} \right] \right. \nonumber \\&\left. -\log \left[ \frac{\Lambda ^2}{m_t^2} \right] \right) \epsilon _{S+P,L}^{ijqt} \simeq \frac{2\alpha _{em}}{\pi } V_{t\beta }\epsilon _{S+P,L}^{ijqt}, \nonumber \\ \epsilon _{CC}^{ij q\beta }\simeq & {} \frac{2\alpha _{em}}{\pi } V_{t\beta } \epsilon _{LQ,L}^{ijqt}. \end{aligned}$$

Or, in a basis with tensor operators,

51 $$\begin{aligned} \epsilon _{CC}^{ij q\beta }\simeq & {} \frac{ g^2 V_{t\beta } }{32 \pi ^2 } \left( \frac{m_W^2}{m_t^2 - m_W^2} \log \left[ \frac{m_t^2}{m_W^2} \right] -\log \left[ \frac{\Lambda ^2}{m_t^2} \right] \right) \epsilon _{S+P,L}^{ijqt}\nonumber \\\simeq & {} - \frac{\alpha _{em}}{\pi } V_{t\beta } \epsilon _{S+P,L}^{ijqt}. \end{aligned}$$

**Table 9:** The last two columns give the numerical value of the coefficient given in the second column, generated by loop containing the top LFV operator given in the first column, of coefficient $2 \sqrt{2} G_F$. The top LFV operator involves the top and the quark $u_q\in \{u,c \}$; the two choices of *q* identify the last two columns. The operators $\mathcal{O}^{S\pm P,L}_{ijqt}$ also induce FCNC operators (involving 2 down-type quarks), but the coefficients are not listed because they are suppressed by two light quark masses

Top op.	Down coeff.	$q=u$	$q=c$
	$\epsilon _{XL}^{ij db}$	$8.0 \times 10^{-8 }$	$7.7 \times 10^{-6}$
$\mathcal{O}^{XR}_{ijqt}$	$\epsilon _{XL}^{ijsb}$	$1.8 \times 10^{-8 }$	$3.4 \times 10^{-5}$
	$\epsilon _{XL}^{ij ds}$	$3.2 \times 10^{-9 }$	$3.1 \times 10^{-7}$
	$\epsilon _{XL}^{ijdb}$	$2.0 \times 10^{- 3}$	$4.6 \times 10^{-4}$
$\mathcal{O}^{XL}_{ijqt}$	$\epsilon _{XL}^{ij sb}$	$4.6 \times 10^{-4 }$	$2.0 \times 10^{-3}$
	$\epsilon _{XL}^{ij ds}$	$8.2 \times 10^{-5 }$	$1.9 \times 10^{-5}$
	$\epsilon _{S \pm P,R}^{ij db}$	$2.4 \times 10^{-5 }$	$5.3 \times 10^{-6}$
$\mathcal{O}^{S\pm P,R}_{ijqt}$	$\epsilon _{S \pm P,R}^{ij sb}$	$5.3 \times 10^{- 6}$	$2.4 \times 10^{-5}$
	$\epsilon _{S \pm P,R}^{ij ds}$	$4.1 \times 10^{-9 }$	$1.8 \times 10^{-8}$
	$\epsilon _{CC}^{ij qb}$	$2.5\times 10^{- 3}$	$2.5\times 10^{-3}$
$\mathcal{O}^{S+ P,L}_{ijqt}$	$\epsilon _{CC}^{ij qs}$	$1.0\times 10^{- 4}$	$1.0 \times 10^{-4}$
	$\epsilon _{CC}^{ij qd}$	$2.0 \times 10^{-5 }$	$2.0\times 10^{-5}$

### C.3 Bounds and cancellations

The numbers in Table 9 can be compared to the bounds on the various $$\epsilon $$s for light quark operators, given in the Tables 6, 7 and 8. For instance, the last column of the third line of Table 9 says that $$\epsilon ^{ijct} _{XR}\frac{4G_F}{\sqrt{2}} (\overline{\ell }_i \gamma _\sigma P_X \ell _j) (\overline{c} \gamma ^\sigma P_R t)$$ generates $$\epsilon _{XL}^{ijds}\frac{4G_F}{\sqrt{2}} (\overline{\ell }_i \gamma _\sigma P_X \ell _j)$$$$ (\overline{d} \gamma ^\sigma P_L s)$$ with

$$\begin{aligned} \epsilon _{XL}^{ijds} = 3.1 \times 10^{-7} \epsilon ^{ijct} _{XR} \lesssim 3.0 \times 10^{-7}\quad \Rightarrow \quad \epsilon ^{e \mu ct}_{XR} \lesssim 1 \end{aligned}$$

where $\epsilon ^{e\mu ds} _{YL} \lesssim 3.0 \times 10^{-7}$ is the upper bound from $K\rightarrow e\bar{\mu }$ given in Table 6, which sets the constraint $\epsilon ^{e \mu ct} _{XR} \lesssim 1$. This bound on the $e \mu ct$ coefficient of the $\mathcal{O}^{XR}$ operators appears in the second row and last two columns of Table 1. In the case of $e\tau c t$ and $\mu \tau c t$ indices, the best bound on the coefficient of the $\mathcal{O}^{XR}$ operators arises from *B* decays. This can be seen because Table 1 gives the origin of the bound as $\mathcal{O}^{LL}_{i\tau db}$, and the bound on these coefficients from Table 6 is from *B* decays.^5^

The bounds of Table 1 implicitly assume that only one LFV top operator is present at a time. This assumption depends on the choice of operator basis, so it is interesting to consider the possibility of cancellations among the $V \pm A$ operators. Many of the bounds arise from pseudoscalar down-type meson decays, which are mediated by an operator of the form $(\overline{e}_i \gamma ^\rho P_X e_j )(\overline{d}_\alpha \gamma ^\rho \gamma _5 d_\beta )$, in which case the bound applies to the combinations $\epsilon ^{ij \alpha \beta } _{XR} - \epsilon ^{ij \alpha \beta } _{XL}$. Since the loop that transforms top operators into down-type quark operators gives different suppression factors for operators involving $t_L$ or $t_R$, we neglect possible cancellations among *XL* and *XR* operators. So we neglect the possibility of cancellations for $V\pm A$ operators.

In obtaining bounds from (pseudoscalar) meson decays, an operator basis that includes tensors is convenient, because the tensors do not contribute [132] to the decays $M \rightarrow \ell _i \bar{\ell }_j$, where *M* is the pseudoscalar meson made of $q_1\bar{q}_2$ and $\ell _i$ are neutrinos or charged leptons. So we quote low-energy bounds in a basis with tensors.^6^

## Appendix D: Single *t* production at HERA

HERA was an $e^\pm p$ collider with $\sqrt{s} = 319$ GeV, where the H1 [111] and ZEUS [112] experiments searched for single top production: $\sigma (e^\pm p \rightarrow e^\pm t X)$. We follow an H1 analysis [111], which is outlined in Sect. D.1. Then in Sect. D.2, we discuss how to translate the results of this analysis into a bound on the LFV process $\sigma (e^\pm p \rightarrow \mu ^\pm t X)$, and in Sect. D.3 we estimate $\sigma (e^\pm p \rightarrow \mu ^\pm t X)$ in the presence of the operators of Appendix A.

### D.1 The H1 analysis

The H1 collaboration set a bound [111]:

52 $$\begin{aligned} \sigma (e^\pm p \rightarrow e^\pm t +X) \le 0.30~\hbox {pb} = \frac{2.3\times 10^{-5}}{m_t^2}\quad \hbox {at }95~\%\hbox { CL}.\nonumber \\ \end{aligned}$$

using 474 pb$^{-1}$ of data, and requiring the top decay $t \rightarrow \mu ^+ \nu b$.^7^ H1 does not give the luminosity in $e^+p$ and $e^-p$, but from [110, 133], we interpret $\ge$228 pb$^{-1}$ of $e^+p$ data and $\ge$205 pb$^{-1}$ of $e^-p$ data at $\sqrt{s}=319$ GeV, so we approximate that half the luminosity was in $e^+p$, and half in $e^-p$.

We would like to use this limit to set a bound on $$ \sigma (e^\pm p \rightarrow \mu ^\pm t +X) $$. This is possible because H1 searched for leptonic decays of the top, $$t\rightarrow \mu ^+ \nu b$$, and it did not require one to observe the $$e^\pm $$. As a first step in a cut-based analysis, H1 required isolated muons with $$p_T> 10 $$ GeV, and missing energy $$E_T \! \! \! \! \! \! \! /~~>12$$ GeV; one found 14 events where they expected $$11 \pm 1.8$$ Standard Model background. Their signal efficiency, for a magnetic moment $$ut\gamma $$ coupling, was 44 %. A $$e^\pm u \rightarrow \mu ^\pm t$$ interaction mediated by our top operators should pass this first set of cuts, so we would like to obtain a limit on the coefficients of our operators from this stage of the analysis. Then H1 required the muon to be positively charged, and in combination with the jet and $$E_T \! \! \! \! \! \! \! /~~$$, to reconstruct to a top. Only four events remain after imposing these cuts, with $$2.1 \pm 0.3$$ expected in the Standard Model. H1’s signal efficiency was 36 % and at this point they obtain the limit $$\sigma (e^+ p\rightarrow (\mu ^+ E_T \! \! \! \! \! \! \! /~~b) e^+ X) \le 0.30$$ pb.

### D.2 Translating to contact interactions

To translate the H1 bound to our contact interactions, we must address various issues.

1. We estimate that the bound on $\sigma (e^+ p\rightarrow (\mu ^+ E_T \! \! \! \! \! \! \! /~~b) e^+ X)$ after the first cut is comparable to the final bound obtained by H1, so we use the limit of Eq. (52). To obtain this curious approximation, we estimate the *t* production cross section that could be excluded at 95 % CL using Poisson statistics for 4 observed events with 2 background expected,^8^ and then we estimate the *t* production cross section that would give a gaussian distribution of events centred $4\sigma$ above 11, that is at 18. We find these cross sections are comparable.
2. Different operators contribute to $e^+ p\rightarrow \mu ^+ t+ X$ and $e^- p\rightarrow \mu ^- t+ X$. Since the luminosity is approximately equally distributed between $e^+ p$ and $e^- p$, we suppose that the bound for an individual operator is $\sigma \lesssim 2\times 0.30$ pb.
3. In the case of our operators, a central $\mu ^\pm$ is produced by the contact interaction, so the top can decay to any lepton (we do not allow for hadronic *t* decays, because they are unlikely to produce $E_T \! \! \! \! \! \! \! /~~\ge 10$ GeV). This means that the upper bound in the cross section is reduced by 1/3, because H1 required that the top decay to $\mu ^+ \nu b$.
4. We need an estimate for the efficiency for our operators. H1 gives a signal efficiency of 44 % after their first cut, in part because they detect muons as a $p_T$ imbalance in the calorimeter, with a 60 % efficiency for detecting a muon of $p_T = 10$ GeV. Since our event has a central muon, like H1’s signal, we take the efficiencies to be the same, and assume this makes our bounds uncertain by a factor ${\sim }2$.

So, in summary, we multiply the H1 bound by 2/3 and impose

53 $$\begin{aligned} \sigma (e^\pm p\rightarrow \mu ^\pm t +X) \le 0.20~\hbox {pb} = \frac{1.5 \times 10^{-5}}{m_t^2}\quad \hbox {at }95~\%\hbox { CL}.\nonumber \\ \end{aligned}$$

### D.3 ${\sigma (e^\pm p \rightarrow \mu ^\pm t +X)}$ in the presence of top operators

The operators of Appendix A induce a differential partonic cross section for $e^\pm u \rightarrow \mu ^\pm t$ that does not diverge for emission at zero angle. We therefore integrate the partonic cross section over all angles (neglecting experimental cuts), to obtain

54 $$\begin{aligned}&\hat{\sigma } (e^\pm u \rightarrow \mu ^\pm t)=|\epsilon _{XX}|^2 \frac{( 1 -m_t^2/\hat{s})^2}{16 \pi m_t^4} \hat{s}\quad \mathcal{O}_{LL},~ \mathcal{O}_{RR} \nonumber \\ \end{aligned}$$

55 $$\begin{aligned}&\quad =|\epsilon _{YX}|^2 \frac{( 1 -m_t^2/\hat{s})^2}{48 \pi m_t^4} \left( \hat{s}+ \frac{m_t^2}{2}\right) \quad \mathcal{O}_{LR},~ \mathcal{O}_{RL} \\&\quad =|\epsilon _{S-P,X}|^2 \frac{( 1 -m_t^2/\hat{s})^2}{192 \pi m_t^4} \left( \hat{s}+ \frac{m_t^2}{2}\right) \quad \mathcal{O}_{S-P,X} \nonumber \\&\quad =\frac{( 1 -m_t^2/\hat{s})^2}{192 \pi m_t^4} \left( [|\epsilon _{S+P,X} + \epsilon _{LQ,X}|^2] \left[ \hat{s}+ \frac{m_t^2}{2}\right] \right. \nonumber \\&\qquad \left. + \mathrm{Re}\{\epsilon _{S+P,X} \epsilon _{LQ,X}\} [\hat{s}-m_t^2] \right) \quad \mathcal{O}_{S- P,X},~ \mathcal{O}_{LQ,X} \nonumber \end{aligned}$$

To obtain the cross section on the proton, we define $x =\hat{s}/s$, and obtain, integrating over the CTEQ10 [134] parton distribution functions:

56 $$\begin{aligned}&\int _{0.33}^{1} \mathrm{d}x x (1-.295/x)^2 f_u(x) = 0.0046,\nonumber \\&\quad \int _{0.33}^{1} \mathrm{d}x x f_u(x) = 0.041 \nonumber \\&\int _{0.33}^{1} \mathrm{d}x (1-.295/x)^2 f_u(x) = 0.0092,\nonumber \\&\quad \int _{0.33}^{1} \mathrm{d}x f_u(x) = 0.096 \end{aligned}$$

where the lower *x* integration limit is estimated to produce, in the centre-of-mass frame, a top of negligible momentum, and a muon of $p_T > 10$ GeV. The $(1-.295/x)^2$ represents the kinematic suppression factor $(1-m_t^2/\hat{s})^2$.

The resulting cross sections are (for $s/m_t^2 = 3.5$):

57 $$\begin{aligned}&\sigma (e^\pm p \rightarrow \mu ^\pm t+X) = 0.0046 s \frac{|\epsilon _{XX}|^2}{16 \pi m_t^4}\nonumber \\&\qquad \Rightarrow \epsilon _{XX}^{e\mu u t}, \epsilon _{XX}^{\mu e u t} < 0.22 \end{aligned}$$

58 $$\begin{aligned}&\quad = \frac{|\epsilon _{XY}|^2}{48 \pi m_t^4} 0.0046( s +m_t^2)\quad \Rightarrow \epsilon _{XY}^{e\mu u t}, \epsilon _{XY}^{\mu e u t} < 0.33 \nonumber \\ \end{aligned}$$

59 $$\begin{aligned}&\quad =\frac{|\epsilon _{S-P,X}|^2}{192\pi m_t^4} 0.0046( s +m_t^2)\nonumber \\&\qquad \Rightarrow \epsilon _{S - P ,X}^{e\mu u t} , \epsilon _{S - P ,X}^{\mu e u t} < 0.66 \nonumber \\&\quad =\frac{|\epsilon _{S+P,X}|^2 + |\epsilon _{LQ,X}|^2}{192\pi m_t^4} 0.0046(s+m_t^2)\nonumber \\&\qquad \Rightarrow \epsilon _{S + P ,X}^{e\mu u t},~ \epsilon _{S + P ,X}^{\mu e u t},~ \epsilon _{LQ ,X}^{e\mu u t},~ \epsilon _{LQ ,X}^{\mu e u t} < 0.66 \end{aligned}$$

where $X,Y \in \{L,R\}$ and the bounds are estimated by imposing the bound of Eq. (53).

### D.4 What about $\epsilon ^{e\tau ut}$ at HERA?

The $\epsilon ^{e\tau ut}$, $\epsilon ^{\tau e ut}$ are poorly constrained by rare decays, whereas $e^\pm u \rightarrow \tau ^\pm t$ could have occurred at HERA. It is unclear to the authors how H1 would have treated a hadronic $\tau$ decay, so we conservatively restrict our study to $\tau \rightarrow e\nu \bar{\nu }$ decays. Allowing any decay of the top, we use the combined H1 bound

60 $$\begin{aligned} \sigma (e^+p\rightarrow e^+ t +X) \le 0.25~\hbox {pb} = \frac{1.9\times 10^{-5}}{m_t^2}\quad \hbox {at }95~\%\hbox { CL},\nonumber \\ \end{aligned}$$

multiplied by $2/BR(\tau \rightarrow e\nu \bar{\nu }) \simeq 12,$ where the 2 is from point 2 of Sect. D.2 above. So $\sigma (e^\pm p\rightarrow \tau ^\pm t +X) \lesssim 3.0~\mathrm{pb}$, and the approximate bounds on $\epsilon ^{e\tau u t}$, $\epsilon ^{\tau e u t}$ are given in Table 1.

## Appendix E: The CMS search for $t\longrightarrow Zq$

The CMS collaboration sets the limit $BR(t\rightarrow Z q) \le 6\times 10^{-4}$ [65], by searching for $t\bar{t}$ production, with one top decaying leptonically, and the other decaying to *Zq* followed by $Z\rightarrow \ell \bar{\ell }$. In this section, we emulate in our framework this published CMS analysis, which searches for a final state similar to ours.

We hence implement in our framework the selection described in [65]. The definition of good jets and isolated leptons is the same as in our analysis described in Sect. 3.3. We select events containing exactly 3 isolated charged leptons (electrons or muons), of which 2 have the same flavour and opposite sign. Events are requested to contain at least 2 jets, and exactly one b-tagged jet, and the missing transverse energy has to be higher than 30 GeV. The invariant mass of the opposite sign and same flavour lepton is required to lie within 78 and 102 GeV/c$^{2}$.

The 2 opposite sign and same flavour leptons are identified as coming from the Z decay, if there is more than one possible lepton pair, the one with invariant mass closest to the Z mass ($m_{Z}$) is chosen. The remaining lepton is associated with the decay of the W and used, with the missing transverse energy, to calculate the neutrino longitudinal momentum with a W mass constraint, as explained in Sect. 3.3. The invariant mass of the W and the b-tagged jet is required to be within 35 GeV/c$^{2}$ of the nominal top mass. The 4-momenta of the 2 leptons coming from the Z are combined with all (non b-tagged) jets in the event. In order for the event to be selected, at least one of these combinations must have a resulting invariant mass within 25 GeV/c$^2$ of the nominal top mass.

All the background processes taken into account in the CMS analysis are considered. In our simulated samples, the expected number of background events after applying the CMS selection is $1.5\pm 0.2$, to be compared with the expectation from simulation of the public CMS analysis, of $3.2\pm 1.2 (\hbox {stat}) \pm 1.5(\hbox {syst})$. This comparison is not meant to be rigorous, as the Delphes emulation of the CMS detector reconstruction is well known to be imperfect, and the samples used here do not include any effect from pileup. Nevertheless, the agreement is good enough to validate our framework as a tool to extract reasonable sensitivity studies. The result of this comparison also motivates the variation of 100 % in the expected background events for the limit calculation, performed in the main study (Sect. 3.4).

The limit setting procedure is validated as well: when using the number of expected events from the public result in our statistical procedure, we obtain an expected upper limit of $BR(t\rightarrow Z q)<0.9\times 10^{-3}$ to compare to the public result $BR(t\rightarrow Z q)<1\times 10^{-3}$.

Having validated our method on expected background allows us to evaluate the constraint set on the $t\rightarrow q \mu ^\pm e^\mp$ branching ratio by the CMS analysis [65]. Indeed, our LFV top events and the CMS analysis share the same overall final state (missing transverse energy, 3 isolated leptons, and 2 jets, of which one is a *b*-jet). Half of our LFV events should give a pair of opposite sign, same flavour leptons, as required in the CMS analysis. However, in our case, they do not come from the *Z*, but, respectively, from the *t* and $\bar{t}$ (see Fig. 2), so they are easily rejected by the CMS requirement that their invariant mass be near $m_Z$. In order to quantify this, we evaluate the efficiency of the CMS event selection on the $t\rightarrow q \mu ^\pm e^\mp$ signal sample, obtaining $\epsilon =0.050\pm 0.005~\%$. This would correspond to an expected limit on LFV in top decays of $BR(t\rightarrow q \mu ^\pm e^\mp )<3.7\times 10^{-3}$, according to the limit setting procedure based on RooStats (a simple rescaling of the published limit with selection efficiencies and cross sections would give a comparable value of $BR(t\rightarrow q \mu ^\pm e^\mp )<4\times 10^{-3}$). Comparing to Eq. (4), we see that this is on the verge of probing LFV top decays mediated by a 4-fermion operator.

## Footnotes

1 The exception is the $S-P$ operator involving $t_L$, whose loop suppression factor would involve two light quark masses.

2 They are given so that in the future, if the experimental bounds improve, the limits can be obtained by simply rescaling the number in the tables. For instance, if the upper bound on $B\rightarrow \tau \ell X$ decays were to improve by two orders of magnitude, the limit on some $\epsilon$s would be divided by 10, and become marginally relevant.

3 The final states where the *W* decays to $\tau ^{-}\bar{\nu _{\tau }}$ are not directly targeted by this search. The fact that such processes, followed by leptonic $\tau$ decays, can pass our selection is taken into account in the signal efficiency, as explained in the following.
